# Uncovering phenotypic inheritance from single cells with Microcolony-seq

**DOI:** 10.1016/j.cell.2025.08.001

**Published:** 2025-09-18

**Authors:** Raya Faigenbaum-Romm, Noam Yedidi, Orit Gefen, Naama Katsowich-Nagar, Lior Aroeti, Irine Ronin, Maskit Bar-Meir, Ilan Rosenshine, Nathalie Q. Balaban

**Affiliations:** 1 The Racah Institute of Physics, Edmond J. Safra Campus, The Hebrew University of Jerusalem, Jerusalem, Israel; 2 Department of Microbiology and Molecular Genetics, Institute for Medical Research Israel-Canada, Faculty of Medicine, The Hebrew University of Jerusalem, Jerusalem, Israel; 3 Pediatrics and Infectious Diseases Division, Shaare Zedek Medical Center, Jerusalem, Israel; 4 Faculty of Medicine, The Hebrew University, Jerusalem, Israel

**Keywords:** single-cell, single-cell heterogeneity, division of labor, bet-hedging, pathogens, EPEC, bacterial differentiation, *S. aureus*, UPEC, bloodstream infection, UTI, epigenetic inheritance, virulence factors, adhesion factor

## Abstract

Uncovering phenotypic heterogeneity is fundamental to understanding processes such as development and stress responses. Due to the low mRNA abundance in single bacteria, determining biologically relevant heterogeneity remains a challenge. Using Microcolony-seq, a methodology that captures inherited heterogeneity by analyzing microcolonies originating from single bacterial cells, we uncover the ubiquitous ability of bacteria to maintain long-term inheritance of the host environment. Notably, we observe that growth to stationary phase erases the epigenetic inheritance. By leveraging this memory within each microcolony, Microcolony-seq combines bulk RNA sequencing (RNA-seq) with whole-genome sequencing and phenotypic assays to detect the distinct subpopulations and their fitness advantages. Applying this directly to infected human samples enables us to uncover a wealth of diverse inherited phenotypes. Our observations suggest that bacterial memory may be a widespread phenomenon in both Gram-negative and Gram-positive bacteria. Microcolony-seq provides potential targets for the rational design of therapies with the power to simultaneously target the coexisting subpopulations.

## Highlights

• Microcolony-seq reveals inherited bacterial heterogeneity in infected human samples
• Distinct coexisting virulence states characterized in EPEC, UPEC, and *S. aureus*
• Phenotypic inheritance is stable for >20 generations but resets at stationary phase
• Microcolony-seq can suggest targets for eliminating coexisting subpopulations

## Graphical abstract

**Figure:**
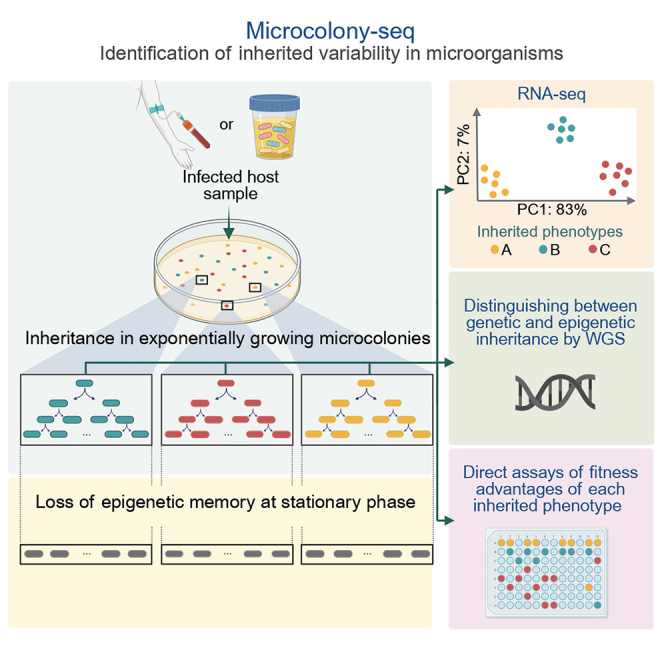

## Teaser

Microcolony-seq systematically uncovers inherited phenotypic diversity directly from infected human samples, revealing that bacteria can retain long-term genetic and nongenetic memory from the host environment. These findings suggest that bacterial memory is widespread and may inform the development of strategies to target the multiple coexisting subpopulations within infections that can evade treatment.

## Introduction

Differentiation in eukaryotes may result in the co-existence of distinct cellular phenotypes that are stably inherited. This phenomenon is evident not only in multicellular tissues but also in unicellular eukaryotes such as yeast^1^^,^^2^ or the malaria parasite.^3^ Phenotypic heterogeneity in bacteria has also become common knowledge since the pioneering work of Novick and Weiner^4^ and has been subsequently observed in many studies revealing single-cell heterogeneity in clonal bacterial populations.^5^^,^^6^^,^^7^^,^^8^^,^^9^^,^^10^^,^^11^ Beyond their contribution to the profound understanding of gene regulation,^12^ studies of phenotypic heterogeneity have also uncovered the far-reaching clinical and evolutionary consequences of bacterial heterogeneity.^13^ Examples include heterogeneity of growth, shown to underlie antibiotic persistence,^14^^,^^15^^,^^16^^,^^17^ and heterogeneity in expression of surface structures, which facilitates infection.^18^^,^^19^

In contrast, some forms of transcriptional heterogeneity may have no major impact on the phenotype. For example, the absence of a specific transcript in a single cell at the time of measurement could represent mere transient noise with no significant phenotypic consequences at the protein level. According to the median number of about 0.1 mRNA transcripts per gene,^20^ such stochastic noise is expected to dominate in single bacteria. In contrast, the stable inheritance of a phenotypic state, unlike such incidental single-cell variability, can be viewed as a differentiation process that may result in stable subpopulations of cells with distinct functions. Therefore, we focus here on inherited heterogeneity.

Inherited heterogeneity in pathogenic bacteria contributes to processes such as host immune evasion,^15^ division of labor,^21^^,^^22^ bet-hedging,^23^ and surface colonization.^24^ General molecular mechanisms of phenotypic inheritance include phase variation by inversion,^25^^,^^26^^,^^27^ DNA methylation,^5^ and positive feedback loops in gene regulation.^28^ A stably inherited phenotypic heterogeneity has been observed in enteropathogenic *Escherichia coli* (EPEC), a human-specific pathogen.^29^^,^^30^^,^^31^ Following transient exposure of EPEC to host conditions, two distinct sizes of genetically identical colonies have been observed, revealing the bistable inheritance of two phenotypes with different growth rates. The memory is maintained during exponential growth but is lost upon reaching the stationary phase. This memory “resetting” step at the stationary phase erases the bistability and results in a unimodal distribution of colony sizes.^30^ A similar differentiation has been observed in the pathogen *Acinetobacter baumannii*, where virulence phenotypes vary by colony opacity.^32^^,^^33^ These bacterial heterogeneities have been identified through visual differences in the colony morphology, a technique tracing back to Robert Koch.^34^ Our goal was to systematically detect inherited variability, without relying on visual cues, using high-throughput RNA sequencing (RNA-seq) and genome sequencing.

Recent advances in cutting-edge methods for single-cell RNA-seq (scRNA-seq) enable bacterial heterogeneity to be revealed by accessing the transcriptome of single bacteria.^35^^,^^36^^,^^37^^,^^38^^,^^39^^,^^40^^,^^41^^,^^42^^,^^43^ Nevertheless, scRNA-seq technologies have several limitations. Typically, RNA is extracted by destructive methods, preventing further analyses of these single cells. Therefore, it cannot be determined whether the variability detected by scRNA-seq is inherited nor whether it arises from mutations or phenotypic processes.^44^ In addition, the phenotypic consequences of the transcriptional heterogeneity and its fitness advantages cannot be directly assessed on the same single cells. These limitations, and the emerging importance of epigenetically inherited variability in microorganisms, prompted us to design Microcolony-seq, a methodology for the systematic identification of inherited variability in bacterial populations. We took advantage of our previous observation that single EPEC bacteria can retain a phenotypic memory of previous exposure to a particular growth condition for many generations.^30^ This memory persists even after bacteria have been shifted to different conditions and plated on solid medium to form colonies, with each retaining the phenotype of its founder bacterium. Thus, the variability of single cells in the original culture results in variability between colonies. Importantly, the amplification of the initial phenotype, from a single bacterium to a colony, allowed the performance of multiple assays on the same subpopulation. By combining RNA-seq, whole-genome sequencing (WGS), and phenotypic characterization, Microcolony-seq distinguishes between genetic and phenotypic inheritance.

First, we applied our method to a clinical isolate of EPEC cultured *in vitro* and identified the unexpected long-term inheritance of numerous differentiation programs, including virulence pathways. The EPEC virulence program, and its dependence on the growth phase, have been extensively studied, revealing key mechanisms for the attachment and infection of host cells (reviewed in Chen and Frankel,^45^ Clarke et al.,^46^ and Vallance and Finlay^47^). Microarray and RNA-seq studies have identified virulence genes by comparing EPEC bacteria grown under different conditions or mutants lacking key regulators.^48^^,^^49^^,^^50^^,^^51^ In contrast, Microcolony-seq dissects transcriptional differences between microcolonies from the wild-type culture grown under the same conditions, thus avoiding spurious environmental or genetic background influence. This enabled not only the identification of heterogeneity and inheritance in the known virulence program activation but also the identification of a virulence factor important for host-cell adhesion.

We then extended the application of our method to analyze variability directly in infected host samples: a urine sample from a patient diagnosed with a urinary tract infection (UTI) caused by uropathogenic *E. coli* (UPEC) and a blood sample from a patient with life-threatening *Staphylococcus aureus* (*S. aureus*) bacteremia. We repeatedly found that the infecting pathogens form a community composed of several stable subpopulations of virulence states, established by both genetic and phenotypic mechanisms. The results of Microcolony-seq highlight the notion that even in “clonal” infections, the host is challenged by a combined attack of distinct subpopulations. Strikingly, in many cases, the memory of the phenotypic states acquired in the host is maintained at the level of the microcolony but is lost upon reaching the stationary phase. Thus, Microcolony-seq provides a unique window into the physiology of single bacteria in the host, especially in infections where the total number of bacteria in the host sample is low, such as in bacteremia. Moreover, the precise mapping of the different coexisting phenotypic states of pathogens reveals the genes that characterize each subpopulation, suggesting targets and drug combinations for the rational design of treatment strategies.

## Results

### Microcolony-seq reveals phenotypically inherited states

**Figure S1:**
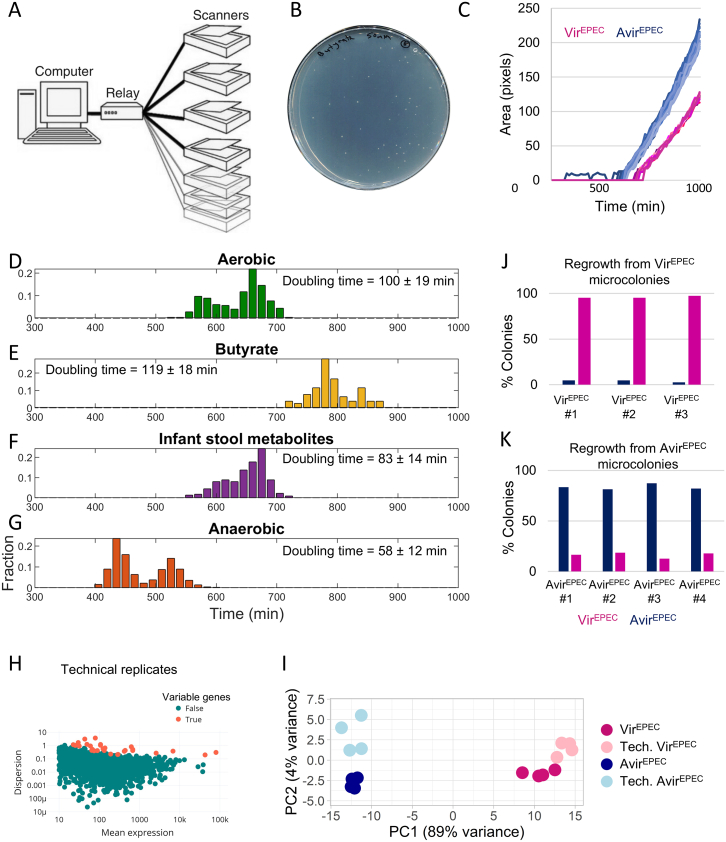
Determination of appearance time of microcolonies for Microcolony-seq methodology, related to Figure 1 (A) Illustration of the ScanLag experimental setup (image adapted with permission from Levin-Reisman et al.^83^). Bacteria are plated on agar plates, placed on office scanners, and incubated in a temperature-controlled room. The software controls the scanners and allows the monitoring of colony appearance by time-lapse imaging. Automated image analysis extracts the appearance time of each colony, as well as its doubling time. (B) A typical LB agar plate with EPEC microcolonies of the size picked for Microcolony-seq methodology (diameter of approximately 500 μm). (C) Growth of individual EPEC microcolonies of LB agar plates monitored by ScanLag.^53^ EPEC bacteria were grown in liquid for 3 h in host-mimicking conditions (DMEM; 37°C) and plated on LB agar plates at 32°C. The plot shows the earlier appearance and faster growth of the Avir^EPEC^ (blue) vs. the Vir^EPEC^ subpopulations (pink). Each growth curve represents one microcolony (*n* = 27 for Avir^EPEC^ and *m* = 12 for Vir^EPEC^ microcolonies). (D–G) Distribution of appearance time of EPEC microcolonies measured by ScanLag.^53^ Growth of EPEC WT bacteria, as described in (C), and plated on agar plates with different conditions: (D) growth on LB agar plates at 32°C (aerobic), (E) growth on LB agar plates supplemented with butyrate (50 mM) at 32°C, (F) growth on LB agar plates supplemented with infant stool metabolites at 32°C, and (G) anaerobic LB plates at 37°C. The bimodality in appearance time is robust to conditions, but the relative fractions of the Vir^EPEC^ and Avir^EPEC^ populations may vary between experiments. The average doubling time of all microcolonies (Vir^EPEC^ and Avir^EPEC^) is indicated for each growth condition, i.e., the time needed for each microcolony to grow from 30 to 60 pixels. (H) Identification of variable genes in technical replicates allows ruling out transcripts prone to technical noise. Dispersion vs. normalized mean expression^54^ for the sequenced reads calculated on tested microcolonies, representing technical replicates. Each dot represents a gene. High dispersion outlier genes, i.e., the variable genes between these replicates, were obtained by dividing a mix of four microcolonies into four separate samples, each undergoing separate extraction, and RNA-seq analyses. The technical variable genes are highlighted in red. Identification of the genes that show inherent variability in technical replicates enabled to exclude them from the list of variable genes in the biological replicates (microcolonies) and, therefore, to better identify the genes that are truly variable due to biological heterogeneity. For example, some tRNAs were found to be technically variable and were excluded from the Microcolony-seq analysis. The technically variable genes can be found in Table S2. (I) PCA plot of Vir^EPEC^ (dark pink, *n* = 4) and Avir^EPEC^ (dark blue, *n* = 4) biological microcolonies, along with their technical replicates (light pink, *n* = 4, and light blue, *n* = 4, respectively). The PCA was generated using variable genes identified in the biological replicates of Vir^EPEC^ and Avir^EPEC^ microcolonies. All samples were normalized together using DESeq2.^54^ (J and K) ScanLag analysis of the regrowth of the individual EPEC microcolonies taken for the aerobic Microcolony-seq experiment (Figure 1). Regrowth of Vir^EPEC^ microcolonies (J) results in mostly Vir^EPEC^ colonies, and regrowth of Avir^EPEC^ microcolonies (K) results in mostly Avir^EPEC^ ones. Results are presented for three Vir^EPEC^ and for four Avir^EPEC^ microcolonies. The percentage of Vir^EPEC^ and Avir^EPEC^ colonies was determined according to the growth time.

**Figure 1:**
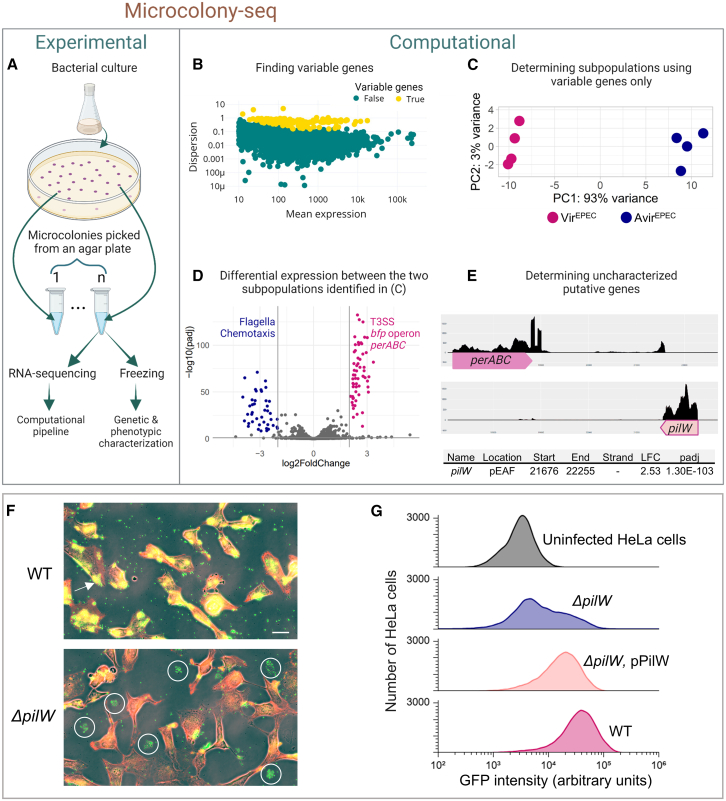
Microcolony-seq identifies phenotypic heterogeneity in EPEC microcolonies (A) Experimental workflow of Microcolony-seq: bacteria are plated on solid medium, microcolonies are sampled as soon as they appear and individually resuspended, with a fraction stored at −80°C for further analyses and the remainder subjected to bulk RNA-seq. Created in BioRender. Romm, R. (2025) https://BioRender.com/u87gclv. (B–D) Microcolony-seq identifies a previously known heterogeneity without relying on colony morphology.^30^ EPEC wild-type (WT) bacteria were grown in liquid for 3 h in host-mimicking conditions (DMEM) and plated on LB agar plates, incubated at 32°C for 15 h, and subjected to the experimental pipeline shown in (A) (*n* = 4 Vir^EPEC^; *n* = 4 Avir^EPEC^). (B) Dispersion vs. normalized mean expression for the sequenced reads calculated on tested microcolonies, representing biological replicates. Each dot represents a gene. High dispersion outlier genes, i.e., the variable genes between the biological replicates, are highlighted in yellow. (C) Principal-component analysis (PCA) plot of the microcolonies using only the variable genes identified in (B) clearly distinguishes between the Vir^EPEC^ and Avir^EPEC^ subpopulations, which correspond to the two morphotypes. (D) Volcano plot illustrating the differential expression analysis^54^ between Vir^EPEC^ and Avir^EPEC^ subpopulations identified in (C), highlighting statistically significant upregulation of EPEC virulence machinery and downregulation of flagellar and chemotaxis genes. Genes colored in pink or blue are statistically significantly increased or decreased, respectively (|log_2_fold change| > 2 and *p*adj < 0.1). (E) Identification of *pilW* as a gene upregulated in Vir^EPEC^ microcolonies. The coverage of the gene expression reads mapped to the region on the pEAF plasmid between *perABC* (on the plus strand) and the *pilW* (on the minus strand) in Vir^EPEC^ is presented. Below are the values of the differential expression as in (D). (F and G) Impaired attachment of *ΔpilW* to HeLa cells. (F) Microscopy of HeLa cells (red:actin) infected with WT EPEC or *ΔpilW* strains. EPEC bacteria marked in green (anti-EPEC antibody). Although the WT strain formed clusters on HeLa cells (marked with an arrow), in the *ΔpilW* strain, clusters were formed on the glass (marked with white circles). Bar size is 10 μm. (G) Quantification of bacterial attachment by flow cytometry analysis of HeLa cells. An inducible plasmid for GFP expression was introduced to WT, *ΔpilW* and *ΔpilW*, pPilW EPEC strains. The bacteria were induced to quantify the number of bacteria attached to HeLa cells. For each sample, the GFP intensity for 60,000 events (corresponding to 60,000 HeLa cells) was measured. The fraction of infected HeLa cells was significantly lower in the *ΔpilW* mutant (*p* = 8.76 × 10^−6^) and restored by complementation of the *ΔpilW* with a plasmid carrying the *pilW* gene (*ΔpilW*, pPilW, *p* = 0.02). Statistical analysis by Student’s t test. See also Figures S1, S2, S3, and S4 and Tables S1 and S2.

As a proof of concept, we applied the Microcolony-seq methodology to the bistable heterogeneity of EPEC bacteria reported by Ronin et al., which was detected thanks to the two distinct colony sizes (named SMALL and BIG).^30^ To test whether Microcolony-seq can systematically detect heterogeneity without prior knowledge of colony size, EPEC bacteria were grown in DMEM at 37°C for 3 h, the host-mimicking conditions typically used to activate virulence in EPEC.^49^^,^^52^ We plated the culture on a Lysogeny Broth (LB) plate and harvested colonies as soon as they became visible to avoid losing inherited states^30^ (see Methods S1 for definition of stability). For this purpose, we utilized the ScanLag setup for automated assessment of colony appearance time (Figures S1A–S1G).^53^ SMALL and BIG colonies were sampled without any washing or concentration steps and bulk RNA-seq was performed. The sampled colonies were typically around 500 μm in diameter (Figure S1B) and were thereafter referred to as microcolonies (STAR Methods). Importantly, a small portion of each microcolony was frozen for further characterization (Figure 1A; Tables S1 and S2).

Inspired by MemorySeq,^55^ which found variable genes in biological replicates of mammalian cells, the first goal of the computational pipeline of Microcolony-seq was to identify the most variable genes among the microcolonies (Figures 1B and S1H). We then clustered the microcolonies based on the expression levels of these variable genes (Figures 1C and S1I). The analysis effectively separated the microcolonies into clearly distinct subpopulations, where the primary variability axis accounts for over 90% of the variation between the two subpopulations of microcolonies. There was a 100% correspondence between the separation of these two subpopulations and the SMALL and BIG colony size morphotypes characterization (Figures S1J and S1K). These results show that Microcolony-seq can identify epigenetically inherited states without relying on colony morphology.

### Microcolony-seq reveals a clear virulence signature of the EPEC bacteria, enabling identification of a critical virulence gene

To explore the differences between the identified subpopulations (Figure 1C), we applied differential expression analysis (Figure 1D). Importantly, the two subpopulations maintained their distinct differentiated states (Figures S1J and S1K), even though they both grew on the same plate. This enabled a direct comparison of the two distinct states, eliminating potential influences stemming from differences in growth conditions or the use of mutant strains, which were previously required for comprehensive investigations of EPEC virulence programs.^48^^,^^49^ When we compared the two identified subpopulations of microcolonies, it became evident that one subpopulation exhibited a significantly higher expression level of the known set of virulence genes.^56^^,^^57^ Therefore, the two distinct subpopulations of microcolonies were subsequently named “Vir^EPEC^” and “Avir^EPEC^,” corresponding to SMALL and BIG morphotypes, respectively. The upregulated genes in the Vir^EPEC^ subpopulation consisted of predominantly annotated EPEC virulence genes (59 out of 60 genes), encoding for components of the type III secretion system (T3SS) and type IV bundle-forming pili (BFP), whereas the downregulated genes in the Vir^EPEC^ subpopulation were associated with motility and chemotaxis (*p*adj < 0.1 and |log_2_fold change| > 2) (Figure 1D; Table S2). A less stringent cutoff (*p*adj < 0.1 and log_2_fold change > 1) for the upregulated genes identified additional putative virulence genes (Table S2).

**Figure S2:**
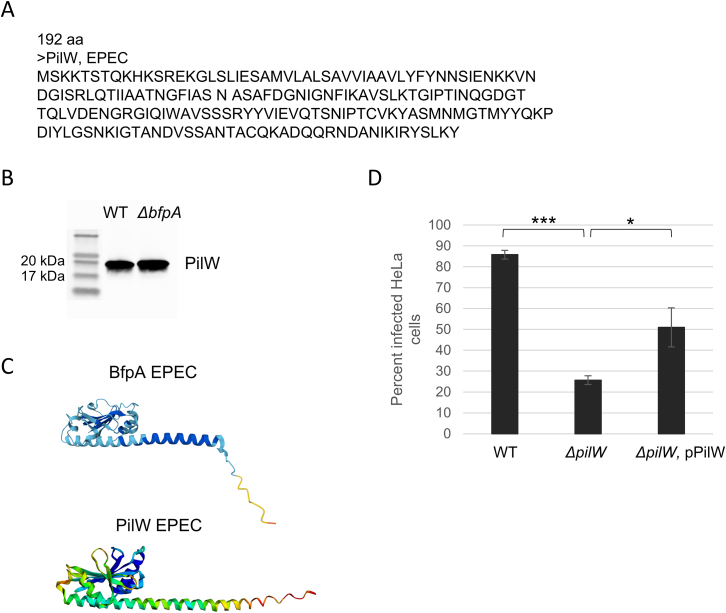
Characterization of PilW: A key virulence gene in EPEC, related to Figure 1 (A) The amino acid sequence of PilW in EPEC. (B) PilW protein is expressed at the expected size in exponentially growing EPEC bacteria. Both the WT EPEC strain and the *ΔbfpA* EPEC strain, in which PilW was FLAG-tagged, were grown to exponential phase in DMEM. PilW was highly expressed in both strains, indicating that PilW is indeed expressed and that its expression is independent of BfpA. (C) Predicted secondary structures for BfpA and PilW were obtained by AlphaFold.^137^ (D) Bacterial attachment to host cells is reduced in the *ΔpilW* strain. Attachment was assessed by flow cytometry using HeLa cells infected for 2.5 h with EPEC strains containing a GFP plasmid (NY11113, NY12134, and NY12136): WT, *ΔpilW*, and *ΔpilW* complemented with a PilW-expressing plasmid. GFP levels from 60,000 infected HeLa cells were measured for each sample using the CytoFLEX X5 flow cytometer. The experiment was performed in triplicate, and data were analyzed and visualized using the Floreada.io platform. A significant reduction in bacterial attachment was observed in the *ΔpilW* strain compared with the WT (*p* = 8.76 × 10^−6^). Attachment was rescued in the *ΔpilW* strain complemented with a PilW-expressing plasmid (*p* = 0.02). The experiment was repeated three times. Statistical significance was determined using a Student’s t test.

Encouraged by the highly significant and coherent virulence gene expression characterization provided by Microcolony-seq, we extended our analysis to unannotated regions in the genome. We identified an open reading frame consisting of 192 codons on the EPEC adherence factor plasmid (pEAF) with a 2.5 log_2_fold increased expression in Vir^EPEC^ (Figure 1E). This putative gene was named *pilW* due to its similarity to the type IV pilus assembly PilW protein in other *E. coli*, *Escherichia albertii*, and *Salmonella enterica* strains. To validate that PilW is expressed, we constructed an EPEC strain with an epitope-tagged PilW and found that a protein of the correct size is produced (Figures S2A and S2B). The predicted structure of PilW is reminiscent of the structure of BfpA, the major pilin of BFP (Figure S2C). BFP expression is associated with two phenotypes: autoaggregation, leading to the formation of bacterial clusters, and attachment of the clusters to the host cell.^58^^,^^59^ Interestingly, while EPEC deleted for *pilW* still self-aggregates, the formed aggregates displayed poor host attachment, which was restored upon *pilW* complementation (Figures 1F, 1G, and S2D). Thus, Microcolony-seq provided a direct comparison between Vir^EPEC^ and Avir^EPEC^ transcriptomes, free from the spurious influence of genetic background or growth conditions, allowing for the detection of a key player in the attachment of EPEC to host cells.

### Microcolony-seq robustly detects inherited states in host-resembling environments

**Figure S3:**
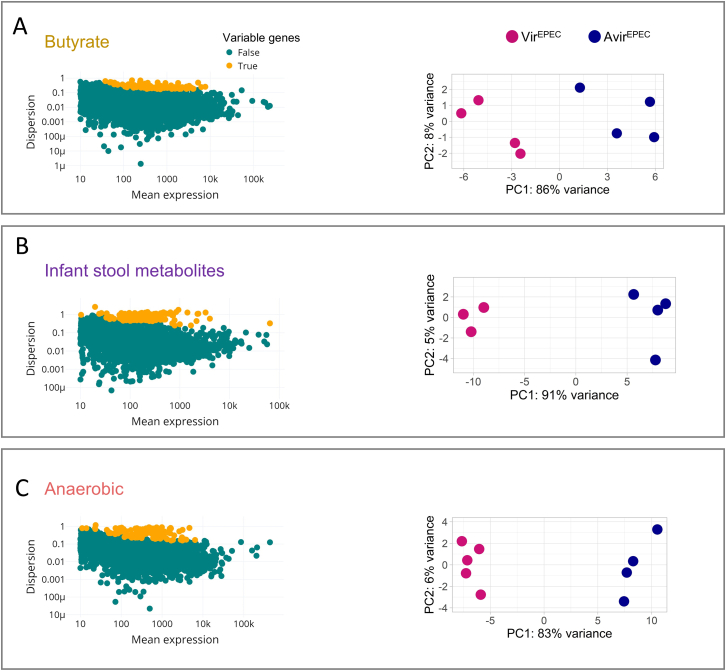
Application of Microcolony-seq on EPEC microcolonies under various host-resembling growth conditions, related to Figure 1 Microcolony-seq was applied to EPEC microcolonies grown under three additional host-resembling conditions. WT EPEC bacteria were grown for 3 h in DMEM at 37°C and plated on LB agar plates supplemented with: (A) butyrate (50 mM), (B) infant stool metabolites at 32°C, or (C) under anaerobic conditions at 37°C. In each condition, *n* = 4 for sampled Vir^EPEC^ and Avir^EPEC^ microcolonies. Left: dispersion vs. normalized mean expression for the sequenced reads in microcolonies grown under each condition. Each dot represents a gene. Highly variable genes, identified as the variable between biological replicates, are highlighted in yellow. Right: PCA of variable genes used to cluster the microcolonies. A clear separation along PC1 was observed between Vir^EPEC^ (pink) and Avir^EPEC^ (blue) microcolonies, accounting for more than 83% of the variance across the three growth conditions. The plots for the aerobic condition appear in Figures 1B and 1C.

**Figure S4:**
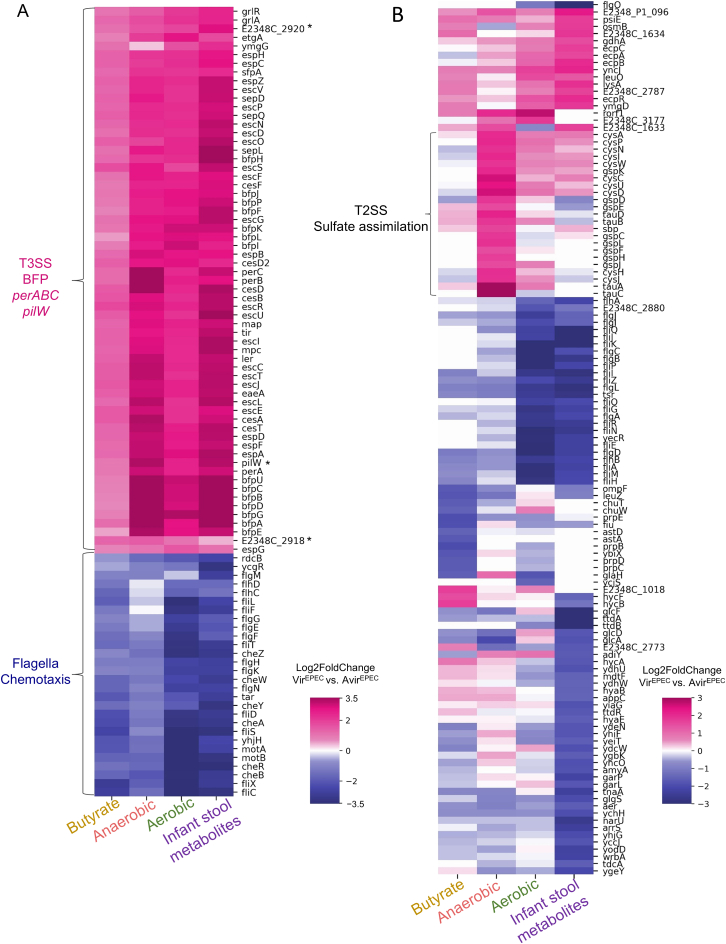
Identification of common as well as distinct virulence programs with Microcolony-seq on EPEC microcolonies under various host-resembling growth conditions, related to Figure 1 (A) A heatmap using the log_2_fold change values of the differential expression analysis^54^ between the Vir^EPEC^ and Avir^EPEC^ microcolonies showing the genes that have been changed (|log_2_fold change| > 1; *p*adj < 0.1) under at least three growth conditions (aerobic, butyrate, infant stool metabolites, and anaerobic). Genes with positive (increased) and negative (decreased) log_2_fold change values are presented in pink and blue colors, respectively. The group of increased genes includes the T3SS, *perABC*, and *bfp* operons, whereas the group of decreased genes includes motility and chemotaxis genes. Genes marked with an asterisk are putative virulence genes and the *pilW* gene identified in this study. (B) Condition-specific differentially expressed genes between Vir^EPEC^ vs. Avir^EPEC^ microcolonies. The heatmap uses log_2_fold change values of significantly changed genes in the comparison between Vir^EPEC^ and Avir^EPEC^ microcolonies (|log_2_fold change| > 1.5; *p*adj < 0.1, DESeq2^54^). Only genes differentially expressed in at most two conditions were included. Positive (pink) and negative (blue) values of log_2_fold change are shown. Genes with low mean expression (below 10 normalized reads) or with non-significant padj (*p*adj > 0.1) are shown in white. T2SS genes (*gsp* operon), sulfate metabolism genes (*cys* operons), and taurine metabolism (*tauABC* operon) are marked to show their increase, predominantly in the Vir^EPEC^ vs. Avir^EPEC^ microcolonies in the anaerobic condition.

To examine whether the states inherited by Vir^EPEC^ and Avir^EPEC^ can be observed in conditions that may be encountered in the host environment and may result in different memory dynamics, we applied Microcolony-seq to the EPEC microcolonies grown on solid media with different supplements. Three conditions were considered: (1) butyrate, part of the microbiome metabolites linked to virulence regulation^60^^,^^61^^,^^62^; (2) whole-stool metabolites derived from an infant’s stool, because EPEC is primarily pathogenic to infants^63^; and (3) anaerobic conditions found in the small intestine, the host niche of EPEC.^45^ Although the growth rates and physiology of the EPEC microcolonies differed between conditions (Figures S1E–S1G), Microcolony-seq repeatedly identified the differentiation into Vir^EPEC^ and Avir^EPEC^ subpopulations (Figures S3A–S3C). A comparison between Vir^EPEC^ and Avir^EPEC^ microcolonies in each growth condition demonstrated common programs, including upregulation of the known EPEC virulence machinery and downregulation of motility and chemotaxis programs (Figure S4A). Putative virulence genes common to several growth conditions were observed, including the *pilW* gene identified in this study (Table S2). In addition, condition-specific pathways were identified, with the most notable example being the type II secretion system (T2SS), which was specifically upregulated only in the anaerobic Vir^EPEC^ microcolonies (Figure S4B). This observation may explain the requirement for T2SS for the virulence of EPEC and related pathogens *in vivo*,^64^^,^^65^ and specifically for the invasion of the anaerobic mucus layer.

### The fitness advantages of the Avir^EPEC^ subpopulation include higher motility and the ability to grow in a high-salt environment

**Figure 2:**
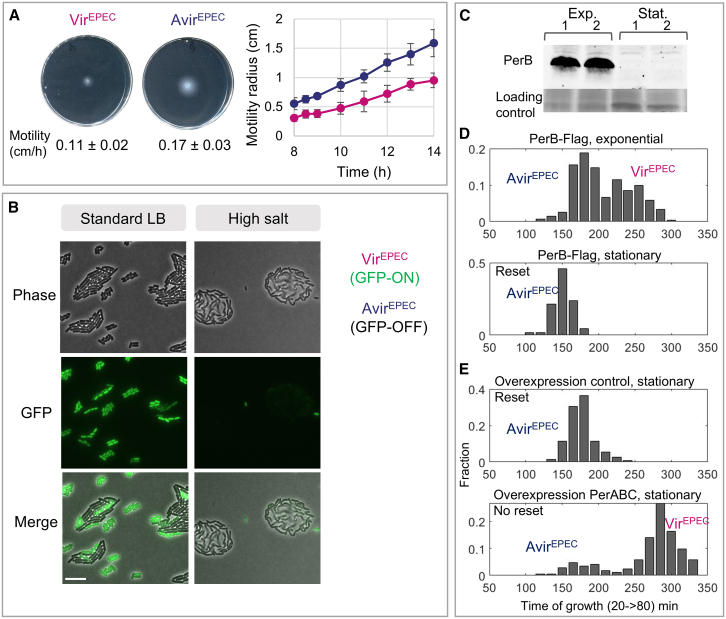
Characterization of the Vir^EPEC^/Avir^EPEC^ differentiation and its reset mechanism at stationary phase (A) The Avir^EPEC^ morphotype is more motile than the Vir^EPEC^ one. Motility comparison between Vir^EPEC^ (*n* = 4) and Avir^EPEC^ (*n* = 4) microcolonies phenotypic analyses on the same microcolonies kept at −80°C from the Microcolony-seq experiment shown in Figure 1. Bacteria from the Avir^EPEC^ (blue) or Vir^EPEC^ (pink) microcolonies were inoculated in the middle of aerobic soft agar motility plates and imaged using the ScanLag setup.^53^ The motility rate was statistically significantly higher in the Avir^EPEC^ microcolonies (*p* = 0.007, by Student’s t test), in agreement with the gene expression results (Table S2). (B) Growth advantage of the Avir^EPEC^ subpopulation under high salt conditions. The analysis was done on fresh microcolonies from a strain bearing the *perABC-GFP* reporter for the Vir^EPEC^ (GFP-ON) and Avir^EPEC^ bacteria (GFP-OFF).^30^ Microscopy images of phase-contrast, GFP fluorescence, and merging of the two channels for EPEC grown on either standard LB plates or on LB plates with high salt concentration. Both Avir^EPEC^ and Vir^EPEC^ morphotypes were able to grow on standard LB plates (images after 2.5 h of growth). However, under high salt conditions, only the Avir^EPEC^ morphotype (GFP-OFF) grew (images after 8 h of growth). The Vir^EPEC^ morphotype (GFP-ON) either switched to the Avir^EPEC^ morphotype (GFP-OFF) or failed to survive. The biofilm-like structures on high salt suggest the secretion of extracellular material (see Video S1). Bar size is 10 μm. (C and D) *per* controls the Vir^EPEC^/Avir^EPEC^ reset at stationary phase. Western blot (C) and ScanLag^53^ (D) analysis were carried-out on the same samples of an EPEC strain with FLAG-tagged PerB grown to exponential phase (3 h growth in DMEM) or to stationary phase (25 h growth in DMEM). (C) PerB is highly expressed during the exponential growth phase and vanishes at stationary phase. Two biological replicates for each growth phase. (D) Bimodal growth quantified in microcolonies plated from exponential phase, corresponding to the Avir^EPEC^ and Vir^EPEC^ subpopulations. Reset to unimodal Avir^EPEC^ growth when plated from stationary phase. (E) Overexpression of PerABC an EPEC strain impairs the reset at stationary phase. The strain with the control plasmid (top) shows the reset at stationary phase as in (D) (bottom), whereas the PerABC overexpression strain (bottom) maintains bimodality at stationary phase without resetting to Avir^EPEC^. See also Figure S5.

To further characterize the identified heterogeneities, we performed phenotypic characterizations of the frozen bacteria preserved from the original microcolonies. Whereas the Vir^EPEC^ state has clear advantages in the host,^45^^,^^66^ we searched for conditions that would unveil the fitness advantages of the Avir^EPEC^ state. In agreement with the significant upregulation of motility and chemotaxis pathways expression measured in Avir^EPEC^ (Table S2), this subpopulation displayed higher motility in quantitative soft agar assays compared with Vir^EPEC^ (Figure 2A). By performing WGS on microcolonies from both Vir^EPEC^ and Avir^EPEC^ subpopulations (Table S3), we confirmed that the phenotypic differences between these subpopulations were not due to genetic differences.

**Figure S5:**
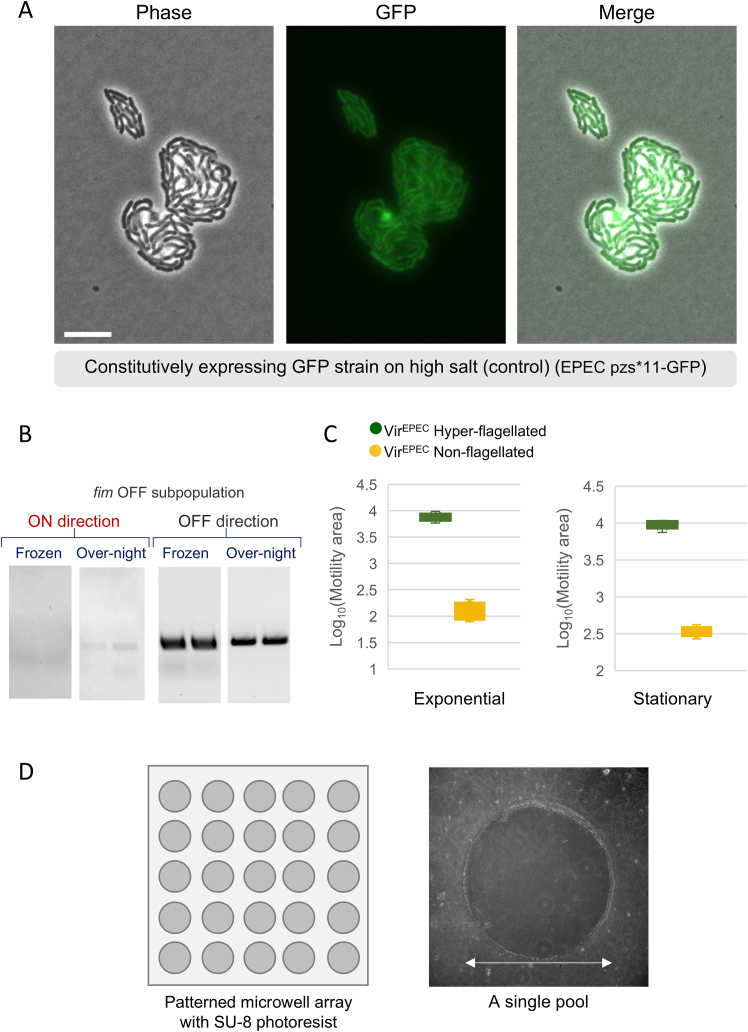
GFP compatibility with high salt growth of EPEC bacteria and motility differences in anaerobic Vir^EPEC^ subpopulations, related to Figures 2, 3, and 4, Video S2, and STAR Methods (A) GFP fluorescence is compatible with growth on high salt. Control showing that GFP fluorescence is compatible with biofilm-like growth on high salt. Constitutively GFP expressing strain EPEC pZS^∗^11-GFP was grown on LB agar pads with 0.7 M NaCl at 37°C. Microscopy images of phase contrast, GFP fluorescence, and merging of both confirm the ability of bacteria to produce GFP and biofilm-like growth under high salt conditions. Micro-Manager software was utilized for time-lapse imaging.^135^ Imaged 8 h after plating. Scale bar: 10 μM. (B) Weak switching of the Vir^EPEC^*fim* OFF state at stationary phase: frozen samples of the same microcolonies analyzed in Figure 3E were grown for 40 h in DMEM at 37°C without shaking, diluted 1:100, and grown for 2 h in DMEM at 37°C without shaking. A comparable number of bacteria of the frozen and after re-growth was used for the PCR. Colonies that were initially only OFF (Figure 3E) now contained a small fraction of bacteria in the ON direction. (C) The difference in motility between the Hyper-flagellated (*n* = 6) and Non-flagellated (*n* = 6) subpopulations in the anaerobic Vir^EPEC^ microcolonies was functional and maintained during the exponential phase (*p* = 0.002 by Wilcoxon rank sum test) as well as in the stationary phase (*p* = 0.002 by Wilcoxon rank sum test). Bacteria from the original microcolonies from the anaerobic condition (Figure 3A) were grown in DMEM in standing conditions at 37°C for 1.5 h (exponential) or 60 h (stationary) and plated on soft agar motility plates. Motility soft agar tests were carried out under aerobic conditions at 37°C. Plates were imaged every 30 min using the ScanLag setup.^53^ The motility area in each time point for each growth phase was quantified using ImageJ software.^82^ The data demonstrated a statistically significant difference in motility between Hyper-flagellated and Non-flagellated subpopulations in both growth phases. (D) A schematic representation (left) of the agar-patterned microwells prepared to observe the motility of the Hyper-flagellated and Non-flagellated EPEC microcolonies at the single-cell level (Video S2). Agar pools were prepared by pouring LB agar on top of silicon wafer patterned with microwells in which bacteria can freely swim. The pattern was done using photolithography with SU-8 photoresist (MicroChemCorp, MA), resulting in a pattern of microwells (100 μm depth and 450 μm diameter). A volume of ∼2 μL of bacteria was placed on a slide and covered by the agar pools. (Right) A representative image of a single pool. Scale bar: 450 μm.

The next set of significantly differentially expressed genes in the Avir^EPEC^ vs. Vir^EPEC^ microcolonies was the group 4 capsule (G4C) genes.^67^ The higher expression levels of these genes pointed to possible growth advantages of the Avir^EPEC^ subpopulation under high salt conditions (Table S2), which leads to capsule expression.^68^ To differentiate at the single-cell level between Vir^EPEC^/Avir^EPEC^ during growth on high salt, we used a strain identical to the wild type previously used but containing a virulence state reporter, namely a transcriptional fusion of GFP to the *per* promoter, as *per* is one of the most highly differentially expressed operon in Vir^EPEC^ (Table S2). We observed that the Avir^EPEC^ bacteria (GFP-OFF) adapt to growth under high salt concentrations, forming biofilm-like colonies with extracellular material separating between the bacteria, consistent with the transcriptome profile of this subpopulation (Table S2). In contrast, the Vir^EPEC^ bacteria (GFP-ON) either switched to the Avir^EPEC^ state (becoming GFP-OFF) or failed to survive (Figures 2B and S5A, control; Video S1).

Video S1. Only the Avir^EPEC^ subpopulation grew under high salt conditions in biofilm-like structures, related to Figure 2 EPEC *ΔperA*, perABC-GFP grown on either standard LB plates (left panel) or on LB plates with high salt concentration (0.7M NaCl, right panel) at 37^°^C. *perA*-GFP reporter was used as a marker of the Vir^EPEC^ morphotype, where *perA* GFP-ON and *perA* GFP-OFF bacteria indicate the Vir^EPEC^ and Avir^EPEC^ bacteria, respectively. Both Avir^EPEC^ (GFP-OFF) and Vir^EPEC^ (GFP-ON) morphotypes were able to grow on regular LB plates (left). However, under high salt conditions, only the Avir^EPEC^ morphotype exhibited growth. The biofilm-like structures suggest the secretion of extracellular material. The Vir^EPEC^ morphotype (GFP-ON) either switched to the Avir^EPEC^ morphotype (GFP-OFF) or failed to survive (right). Scale bar: 10 μM.

### Reset of the Vir^EPEC^ to Avir^EPEC^ at stationary phase

This switching of the Vir^EPEC^ to Avir^EPEC^ upon salt stress is reminiscent of the reset of the Vir^EPEC^/Avir^EPEC^ bimodality to Avir^EPEC^ when reaching the stationary phase.^30^ First, we note that Ronin et al. showed that the expression of PerAB is necessary for establishing the Vir^EPEC^/Avir^EPEC^ bimodality and for the memory of the Vir^EPEC^ state.^30^ To gain insight into the resetting of the Vir^EPEC^/Avir^EPEC^ bimodality, we engineered a PerB-tagged strain and used it to monitor the protein level at the different phases of growth as a proxy for the expression of the *perABC* operon. A significant reduction in PerB level was observed at the stationary phase (Figure 2C), in agreement with previous results^69^ and consistent with the reset of the Vir^EPEC^ state at the stationary phase (Figure 2D). To test whether this significant reduction in *perABC* expression at the stationary phase is the main reason for the erasure of the memory, we overexpressed the PerABC proteins at stationary phase from a plasmid. Strikingly, we observed that the memory erasure of the Vir^EPEC^ phenotype was impaired (Figure 2E), demonstrating that PerABC protein overexpression is sufficient for the maintenance of the memory. This indicates that the downregulation of the *per* promoter at the stationary phase is involved in memory erasure of the Vir^EPEC^ state. As PerA was shown to positively autoregulate its own expression,^70^ this positive feedback loop may lead to bistability, similar to the bistability observed in positive feedback loops in the *lac* or *ara* operons in *E. coli*.^4^^,^^28^^,^^71^ Nonetheless, the detailed mechanism of the resetting at stationary phase involving the PerABC operon remains to be further explored.

### Uncovering an unexpected stability of the *fim* ON state in EPEC

**Figure 3:**
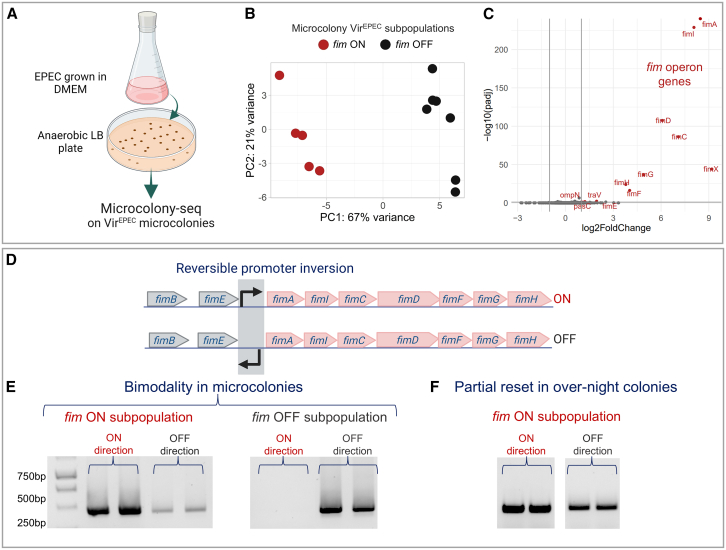
Phenotypic heterogeneity in *fim* expression within the anaerobic Vir^EPEC^ microcolonies (A) EPEC WT bacteria were grown for 3 h in DMEM, plated on anaerobic LB plate, and incubated at 37°C for 12 h. Microcolony-seq was performed on microcolonies of the Vir^EPEC^ morphotype (*n* = 12) with uniform size and morphology. (B) PCA of anaerobic Vir^EPEC^ microcolonies using only the variable genes. Separation between microcolonies with high *fim* expression (red, *n* = 5) and low *fim* (black, *n* = 7) are identified along PC1. (C) Volcano plot of the differential expression analysis^54^ between the two subpopulations based on PC1 separation. Colored gene names present the statistically significant upregulated genes (log_2_fold change > 1; *p*adj < 0.1), revealing higher expression of all *fim* genes in the red subpopulation compared with the black one. (D) Schematic representation of the *fim* operon, highlighting the region undergoing reversible phase variation at the *fimA* promoter. Created in BioRender. Romm, R. (2025) https://BioRender.com/w7a4fjg. (E) *fim* operon state maintenance in anaerobic EPEC microcolonies: PCR results with two sets of primers for the ON and OFF states demonstrated that microcolonies were predominantly either in the *fim* ON or OFF state (two biological replicates of each are shown). Analyses were performed on the same microcolonies analyzed by Microcolony-seq and kept at −80°C. (F) Partial reset of the *fim* ON state at stationary phase: frozen samples of the same microcolonies analyzed in (E) were grown overnight. Colonies that were initially *fim* ON (E) now also contained bacteria in the *fim* OFF direction. See also Figure S5 and Table S3.

Motivated by the comprehensive picture of the differentiation between Vir^EPEC^ and Avir^EPEC^ subpopulations obtained by Microcolony-seq, we asked whether our methodology might reveal further differentiation within the Vir^EPEC^ subpopulation. We thus applied Microcolony-seq to 12 morphologically identical Vir^EPEC^ microcolonies grown under anaerobic conditions, as found in the small intestine environment (Figure 3A). The computational pipeline revealed distinct subpopulations within the Vir^EPEC^ microcolonies (Figure 3B). Differential expression analysis of the subpopulations, defined by their separation along PC1, unveiled significant differences in the expression levels of genes within the *fim* operon, encoding for type 1 fimbriae virulence factor.^72^ These subpopulations exhibited either remarkably high or exceptionally low expression (Figure 3C; Table S3), resembling the reversible ON and OFF phases of the *fim* promoter inversion system.^26^^,^^73^ This observation implied that each microcolony originated from a cell in a different *fim* phase, which was maintained for at least 20 generations, i.e., during growth from the founder bacterium to microcolony size. We analyzed further the frozen EPEC microcolonies kept from the Microcolony-seq of anaerobic Vir^EPEC^ microcolonies. Primers designed to identify the ON and OFF *fim* phase variation directions in a polymerase chain reaction (PCR) enabled the detection of each *fim* state.^73^ The results clearly demonstrated that the variation in *fim* gene expression was due to a phase variation state that was maintained at the microcolony level, i.e., each microcolony essentially contained bacteria only in one of the *fim* states (Figures 3D and 3E). WGS confirmed these observations and provided quantification for the proportion of each *fim* state (in *fim* ON microcolonies: 87%–94% ON; in *fim* OFF microcolonies: 100% OFF) (Table S3). Using a simple model for *fim* state inheritance to quantify the switching rate (Methods S1), we showed that the ON to OFF switching rate in the microcolonies is orders of magnitude lower than previously measured for *E. coli*.^74^^,^^75^^,^^76^ The switch to *fim* ON may be related to the anaerobic conditions, as no *fim* ON microcolony was detected in aerobic conditions (Table S2). Even after overnight growth, the inheritance of the *fim* state was only partially lost (Figures 3F and S5B).

### Stability of the Hyper-flagellated subpopulation in Vir^EPEC^ microcolonies is due to genetic mutations

**Figure 4:**
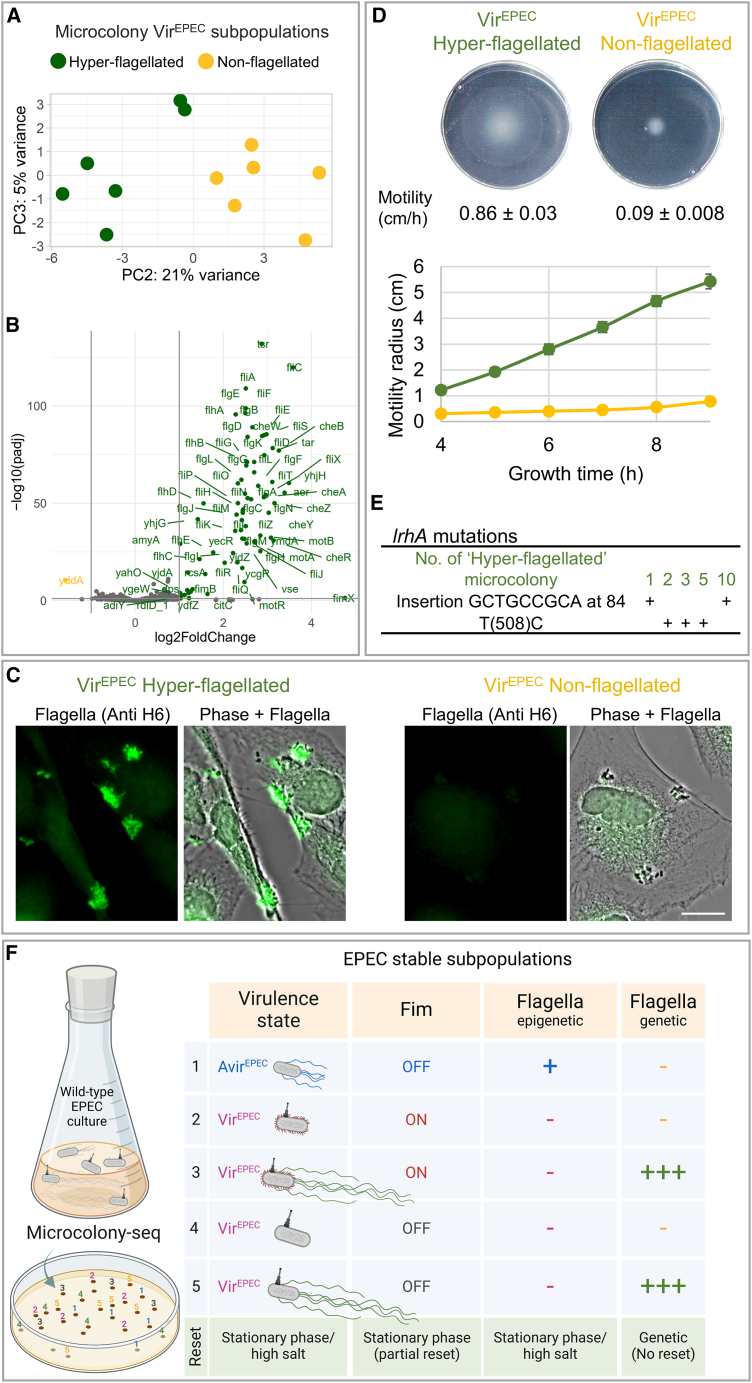
Genetic heterogeneity in flagellar repressor revealed in anaerobic Vir^EPEC^ microcolonies (A) PCA plot of anaerobic Vir^EPEC^ microcolonies using only the variable genes, presenting the PC2 and PC3 axes, color coded by PC2 values. The Vir^EPEC^ Hyper-flagellated (*n* = 6) and Vir^EPEC^ Non-flagellated (*n* = 6) subpopulations are depicted in green and yellow, respectively. (B) Volcano plot of the differential expression analysis between the two subpopulations based on PC2 separation.^54^ Colored gene names present the statistically significant upregulated genes (|log_2_fold change| > 1; *p*adj < 0.1), revealing major motility gene upregulation in the green subpopulation compared with the yellow one. (C) Transcriptional differences in flagellar genes translate to pronounced differences in flagella expression between the Vir^EPEC^ Hyper-flagellated and Vir^EPEC^ Non-flagellated subpopulations. Bacteria from both subpopulations were used for infection of HeLa cells. Flagella were stained with an anti H6 antibody (green). Notably, the Vir^EPEC^ non-flagellated bacteria had a very low expression of flagella. Bar size is 10 μm. (D) Motility differences between the Vir^EPEC^ Hyper-flagellated and Vir^EPEC^ Non-flagellated subpopulations. Aerobic soft agar motility plates were imaged with ScanLag^53^ and quantified using ImageJ.^82^ Analyses were performed on the same microcolonies analyzed by Microcolony-seq and kept at −80°C. The motility rate was statistically significantly higher in the Vir^EPEC^ Hyper-flagellated (*n* = 3) vs. Vir^EPEC^ Non-flagellated (*n* = 3) microcolonies (*p* = 1.8 × 10^−6^, by Student’s t test), in agreement with the gene expression results (Table S3). (E) The Hyper-flagellated variants arise from high-frequency mutations in a flagellar repressor. WGS results uncover mutations in the coding sequence of *lrhA*, a repressor of FlhDC. (F) Schematic representation of all coexisting subpopulations detected by Microcolony-seq in EPEC microcolonies. Three types of heterogeneity (Vir^EPEC^/Avir^EPEC^, *fim* ON/OFF, and Vir^EPEC^ Hyper-flagellated/Non-flagellated) generated five bacterial phenotypes. Stability of inheritance was mapped. Created in BioRender. Romm, R. (2025) https://BioRender.com/tmtvfy0. See also Figure S5, Table S3, and Video S2.

An additional separation was observed along PC2 (Figure 4A). Differential expression analysis revealed a significant upregulation of flagellar and chemotaxis genes in one subset of the Vir^EPEC^ microcolonies compared with the other (Figure 4B), suggesting a hypermotile phenotype. This observation was puzzling in light of the typical downregulation (Figure S4A) and poor motility in the Vir^EPEC^ subpopulation, as quantified by the soft agar assay (Figure 2A). Here, we observed differential expression of motility genes that even exceeds that of the Avir^EPEC^ subpopulation (Table S3). This higher expression also resulted in high expression of flagella (Figure 4C) and a phenotypic hypermotility in soft agar (Figure 4D), with 5- and 9-times higher motility than the Avir^EPEC^ and Vir^EPEC^, respectively. To understand the paradoxical observation of hypermotility in some of the Vir^EPEC^ microcolonies (Figures 4C and 4D), we first investigated the stability of the phenotype. In contrast to the loss of memory and reset observed in the bistability of the Vir^EPEC^/Avir^EPEC^ subpopulations, the bimodality of the hypermotility state was extremely stable and no reset was observed at stationary phase (Figure S5C). By directly observing the swimming of single cells under the microscope using custom-made microscopic pools (Figure S5D), we determined that the Hyper-flagellated and the Non-flagellated Vir^EPEC^ phenotypes were each uniform at the single-cell level (Video S2). WGS of microcolonies revealed mutations within the *lrhA* gene in the Hyper-flagellated subpopulation, including a microsatellite DNA tandem repeat known to lead to high mutation rates^77^ (Figure 4E). These mutations lead to hypermotility, likely due to the release of LrhA-mediated flagellar repression.^78^ The high rate of mutations observed suggests that *lrhA* is a hotspot for mutations, as observed in *E. coli* K12.^79^^,^^80^ Interestingly, mutations in *lrhA* have been linked to highly virulent EPEC strains in children, resulting in death,^81^ suggesting the importance of the Hyper-flagellated Vir^EPEC^ subpopulation for virulence. Notably, without the ability to analyze the genomes of the microcolonies retrospectively, the high rate of emergence of hypermotile microcolonies, without apparent selection pressure for motility, would have been attributed to phenotypic heterogeneity.

Video S2. Single-cell motility is uniform in each Vir^EPEC^ microcolony, either high motility (left) or no motility (right), related to Figure 4 Motility of bacteria from both subpopulations was assessed by microscopy. Left: bacteria from the Hyper-flagellated Vir^EPEC^ subpopulation (microcolony # 1); right: bacteria from the Non-flagellated Vir^EPEC^ subpopulation (microcolony #9). Note that the slight motion in the Non-flagellated Vir^EPEC^ bacteria is due to Brownian motion, not to motility. Scale bar:10 μM.

Taken together, the application of Microcolony-seq to a group of morphologically identical Vir^EPEC^ microcolonies revealed distinct inherited heterogeneous gene expression programs involving *fim* and flagellar gene expression, which, together with the Avir^EPEC^ subpopulation, account for five distinct stable and coexisting subpopulations (Figure 4F).

### Genetic and phenotypic heterogeneity in a patient with a UTI

**Figure 5:**
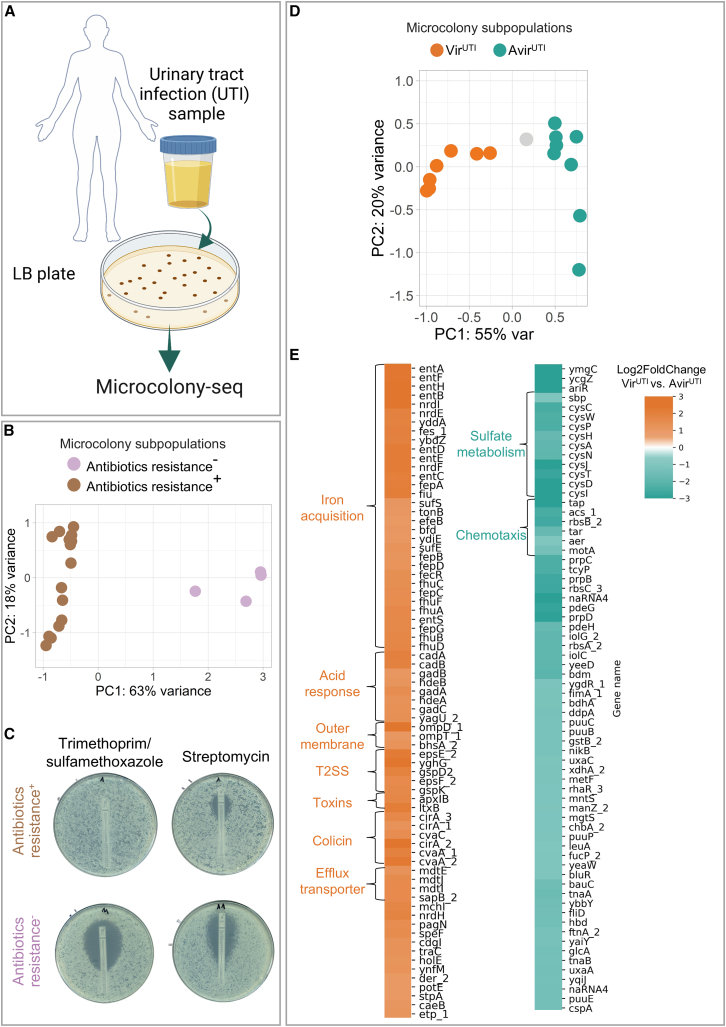
Unraveling bacterial heterogeneity in a clinical UTI using Microcolony-seq (A) Urine obtained from a UTI patient infected with UPEC was spread on LB agar and growth was monitored by ScanLag.^83^ Microcolonies (*n* = 20 for biological and *n* = 4 for technical) were picked as soon as they appeared, resuspended, and analyzed by Microcolony-seq. Created in BioRender. Romm, R. (2025) https://BioRender.com/ah5epdp. (B) PCA utilizing the variable genes (Figure S6C) reveals two UTI subpopulations: those that lost the antibiotic resistance cluster encoding for resistance genes to streptomycin, sulfonamide, and tetracycline (*n* = 4, purple) and those expressing it (*n* = 16, brown), Table S4. WGS performed on the same microcolonies (Table S5) attributes these differences to genetic changes. (C) E tests with trimethoprim/sulfamethoxazole and streptomycin antibiotics performed on the same UTI microcolonies analyzed by Microcolony-seq and kept at −80°C provided confirmation of the heterogeneous resistance levels predicted from the genetic analyses for the Antibiotic resistance^+^ and Antibiotic resistance^−^ subpopulations. (D) PCA utilizing the variable genes (Figure S6C), including only the Antibiotic resistance^+^ subpopulation. PC1 differentiated between two additional subpopulations: Vir^UTI^ (*n* = 7, orange) and Avir^UTI^ (*n* = 8, turquoise). One microcolony indicated in gray could not be attributed to any subpopulation. The Vir^UTI^/Avir^UTI^ separation was shown to be due to phenotypic changes (Table S5). (E) Differential expression analysis between Vir^UTI^ and Avir^UTI^ subpopulations. The heatmaps display genes with |log_2_fold change| > 1 and *p*adj < 0.1, excluding uncharacterized genes. Upregulated genes (orange) include virulence factors like iron acquisition, acid response, T2SS, toxins, and colicins. Downregulated genes (turquoise) include sulfate metabolism and chemotaxis processes. The upregulation of acid response genes suggests that Vir^UTI^ microcolonies maintained memory of the host’s acidic urine (pH 5.0). See also Figure S6 and Tables S4 and S5.

**Figure S6:**
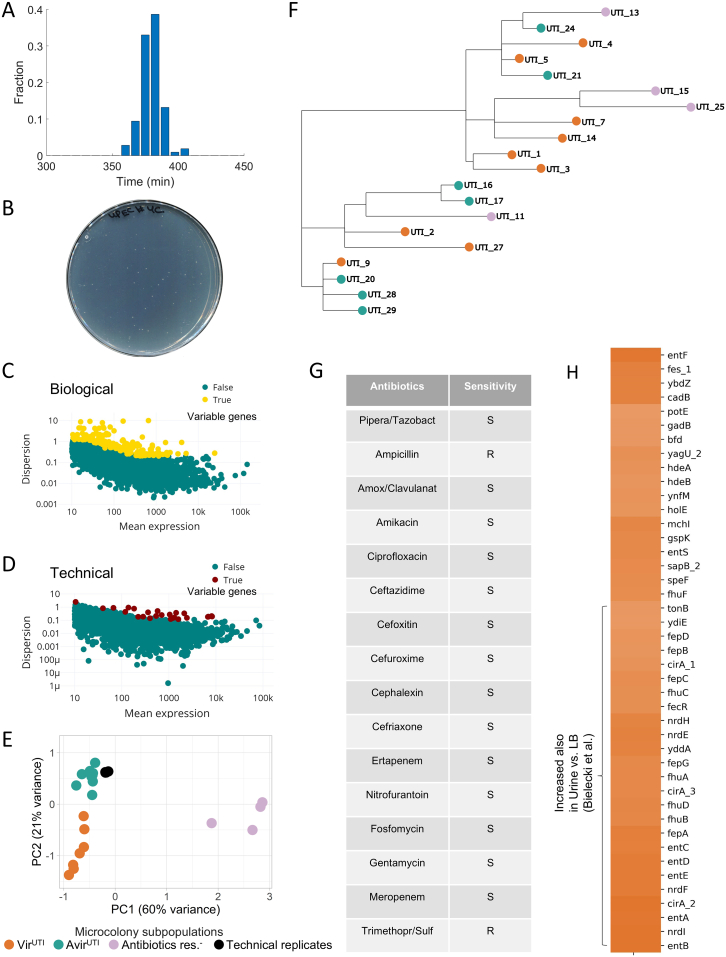
Additional information on the human urine sample (UTI) analyzed by Microcolony-seq, related to Figures 5 and 6 (A) Urine of a patient with a UTI of UPEC bacteria was plated on LB agar plate and incubated at 37°C for 8 h. Appearance time of UTI microcolonies was monitored by ScanLag.^53^ Unimodal appearance time distribution was observed. (B) An agar plate with UTI microcolonies displaying the typical size of microcolonies used for Microcolony-seq. (C) Identification of variable genes in UTI microcolonies (*n* = 20). Variable genes were identified based on gene dispersion (according to Equation 1) and normalized mean expression.^54^ Each dot on the graph represents a gene, with variable genes highlighted in yellow. (D) Identification of variable genes in technical replicates allows ruling out transcripts prone to technical noise. Dispersion (according to Equation 1) vs. normalized mean expression^54^ for the sequenced reads calculated on technical replicates. These technical replicates were obtained by picking and resuspending four UTI microcolonies together in a single Eppendorf tube. The mixture was then divided into four Eppendorf tubes, each of which was subjected to RNA-seq extraction and analysis separately. Each dot represents a gene. Technical variable genes are highlighted in red. (E) A PCA plot of UTI microcolonies, including technical replicates (indicated in black). The PCA was performed using the variable genes identified across biological replicates. All microcolony counts were normalized together using DESeq2.^54^ Colors (orange, turquoise, and purple) correspond to subpopulations identified in the PCA plots (Figures 5B and 5D). Orange denotes Vir^UTI^, turquoise represents Avir^UTI^, and purple signifies antibiotic resistance cluster loss. (F) A phylogenetic tree, constructed from WGS data of UTI microcolonies analyzed by Microcolony-seq, illustrates genetic relationships. Colors as in (E). All microcolonies belong to the same clone. The shortest edge represents one genetic difference. For example, UTI_9 and UTI_20 differ by only one single nucleotide polymorphism (SNP). All detected genetic changes were included in Table S5. Note that the phenotypic clusters (orange and turquoise) are not due to genetic clustering. (G) Antibiotic susceptibility testing obtained from the clinic on the same UTI sample used for the Microcolony-seq experiment. The infecting UPEC bacteria were classified as resistant to ampicillin and to trimethoprim/sulfamethoxazole. Our results showed that a clinical test typically performed on a single colony may not reflect the resistance pattern of the sample (Figure 5C). Only part of the bacteria within the sample were actually resistant to trimethoprim/sulfamethoxazole. S, sensitive; R, resistant. (H) A heatmap based on log_2_fold change values of the DESeq2 analysis^54^ highlights differential expression (log_2_fold change > 1; *p*adj < 0.1) between Vir^UTI^ and Avir^UTI^ microcolonies (Figure 5D). The upregulated genes were marked in orange. We found a significant overlap with a gene set identified in a study comparing bulk RNA-seq data from urine samples of UTI patients to bacteria grown in LB medium.^85^ Our analysis indicated that the Vir^UTI^ microcolonies, despite being grown on LB, kept a memory of the host environment. Only genes with matching names in both datasets were included in this comparison.

In order to test whether inherited heterogeneity from host conditions can be detected in infected host samples, Microcolony-seq was first applied to urine collected from a UTI patient and plated on LB agar. Microcolony-seq applied to 24 morphologically uniform microcolonies (Figures 5A and S6A–S6E) initially revealed two main subpopulations, which were found to differ in the expression of an antibiotic resistance gene cluster including streptomycin, sulfonamide, and tetracycline (Figure 5B; Table S4). WGS of each microcolony revealed that all microcolonies derived from the same UPEC strain, with only a few variations (Figure S6F; Table S5). Microcolonies harboring the resistance cluster genes were indeed resistant to the corresponding antibiotics in E test assays (Figure 5C). Clinical tests on a single isolate revealed resistance to trimethoprim/sulfamethoxazole (Figure S6G). This indicates a risk: selecting only one susceptible isolate for testing could lead to prescribing a treatment ineffective against the patient’s actual infection. Therefore, to more accurately determine drug susceptibility, testing multiple colonies from the same patient is recommended.

Two additional subpopulations of microcolonies were detected using the Microcolony-seq pipeline (Figure 5D) that did not correlate with genetic changes (Table S5), suggesting a phenotypic inheritance phenomenon. These subpopulations differed in the expression levels of major virulence programs such as iron metabolism,^84^ T2SS, colicin, and toxins (log_2_fold change > 1, *p*adj < 0.1) (Figure 5E). We found a highly significant overlap between the differentially expressed genes detected here and those linked to host conditions in a previous comprehensive study of whole-urine analysis^85^ (*p* value 4.6 × 10^−26^) (Figure S6H). We named these subpopulations Vir^UTI^ and Avir^UTI^. In addition to the known virulence genes, we detected elevated expression of the acid response genes in Vir^UTI^ compared to Avir^UTI^, pointing to a memory of an acidic environment. Interestingly, the pH of the patient’s urine was acidic (pH = 5.0), providing further support that these microcolonies maintained a memory of the host environment. Also upregulated in Vir^UTI^ was *ompD*, which has been linked to recurrent UTIs,^86^ whereas sulfate assimilation and chemotaxis genes were downregulated (Figure 5E; Table S4).

### Iron variability leads to functional differences with short-term inheritance

**Figure 6:**
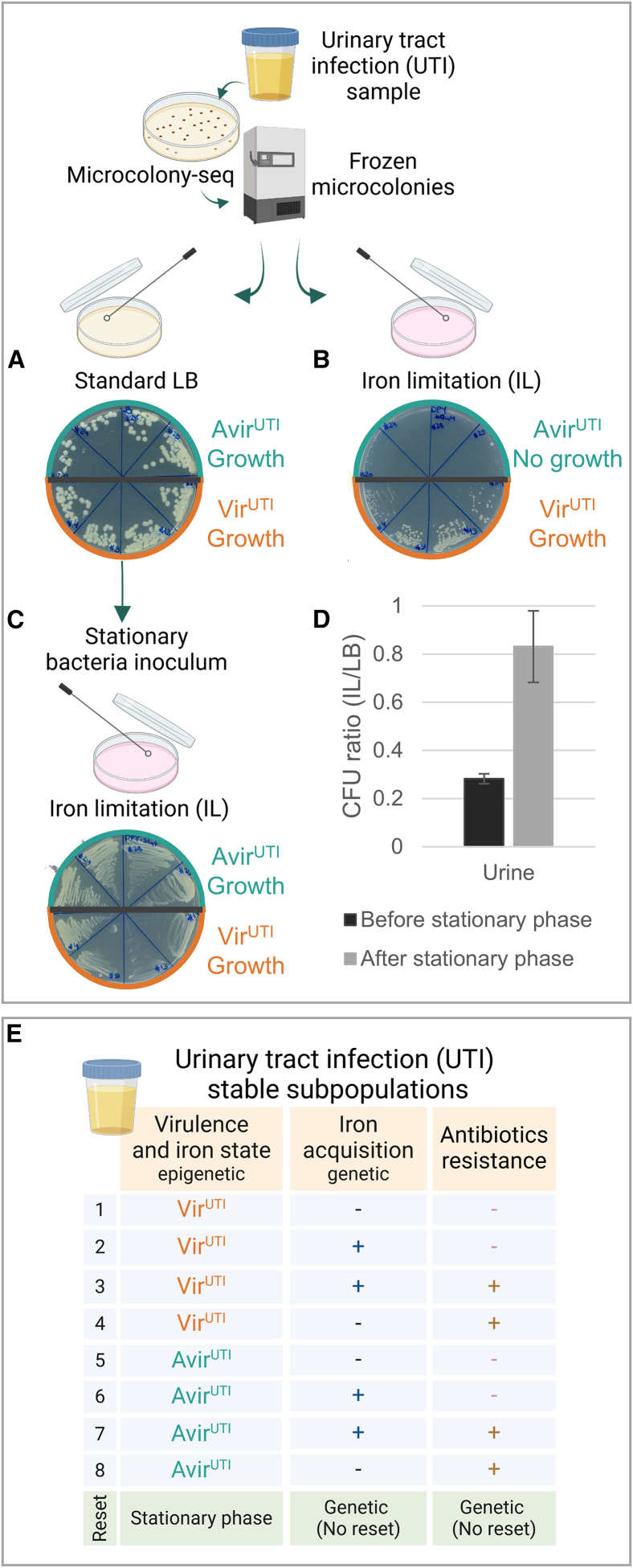
Probing the stability of inheritance of the different subpopulations in the UTI sample All analyses were performed on the same microcolonies analyzed by Microcolony-seq (Figure 5) and kept at −80°C. (A–C) Phenotypic consequences of iron pathway expression heterogeneity in Vir^UTI^ (*n* = 4, high iron expression, orange) and Avir^UTI^ (*n* = 4, low iron expression, turquoise) microcolonies. Microcolonies were plated on standard LB and on IL LB agar. All microcolonies grew similarly on standard LB (A), but only Vir^UTI^ grew under iron limitation (B), revealing the hypersensitivity of Avir^UTI^ to IL. Created in BioRender. Romm, R. (2025) https://BioRender.com/b43y573. (C) The distinction between Vir^UTI^ and Avir^UTI^ phenotypes was lost after growth to stationary phase on standard LB, as both subpopulations could grow on IL conditions, indicating a phenotypic switch. (D) Hypersensitivity to iron limitation was observed in the original urine but not after the bacteria were grown to stationary phase, as quantified by the ratio of CFUs that grew under IL vs. CFUs on LB. The experiment was repeated twice. Mean values of three biological replicates were included. The standard deviation is indicated. (E) Schematic representation of all coexisting subpopulations detected by Microcolony-seq in the UTI clinical sample, showing epigenetic heterogeneity in virulence. This diversity provided a fitness advantage under IL conditions and reset at stationary phase. Genetic and epigenetic heterogeneity resulted in eight different subpopulations. Created in BioRender. Romm, R. (2025) https://BioRender.com/b32o015. See also Figure S6.

Iron acquisition is crucial for bacterial survival and pathogenicity within the host environment.^87^ The heterogeneity in the expression of iron acquisition pathways revealed by Microcolony-seq (Figure 5D; Table S4) suggested that the Vir^UTI^ and Avir^UTI^ microcolonies may differ in their ability to grow under iron-limiting (IL) conditions. To test this prediction, we grew bacteria directly from the frozen samples of the original microcolonies used in the Microcolony-seq experiment. We cultured the bacteria on standard LB plates or on LB plates supplemented with 2,2′-dipyridyl, resulting in IL conditions. Whereas all bacteria grew similarly on standard LB (Figure 6A), only a subset of the samples grew on the IL plates (Figure 6B). In agreement with the Microcolony-seq transcriptome profile, the microcolony subset hypersensitive to iron limitation was solely composed of the Avir^UTI^ subpopulation (Figure 6B). To test whether the iron hypersensitivity phenotype existed in the host sample, we used the original urine sample to quantify the ratio of colony-forming units (CFUs) in IL vs. standard LB. We found that most of the bacteria (72%) were unable to grow on the IL plates. Notably, whereas the memory of the iron metabolism differentiated state was maintained when grown in microcolonies, this heterogeneity was erased after overnight incubation (Figure 6C). In agreement with the understanding that the hypersensitive phenotype originates in the host and is reset by the stationary phase, overnight culture of the urine erased the hypersensitivity to iron limitation (Figure 6D). Taken together, these results show that this phenotype originates in the host, is inherited at the level of the Avir^UTI^ microcolony, and is lost upon entry into stationary phase *in vitro*. Additional genetic variability, unrelated to the phenotypic heterogeneity described above, was found in genes encoding iron siderophores. The various subpopulations identified in UPEC in the urine are summarized in Figure 6E.

### Microcolony-seq reveals that different subpopulations express different antigens in an *S. aureus* BSI

**Figure 7:**
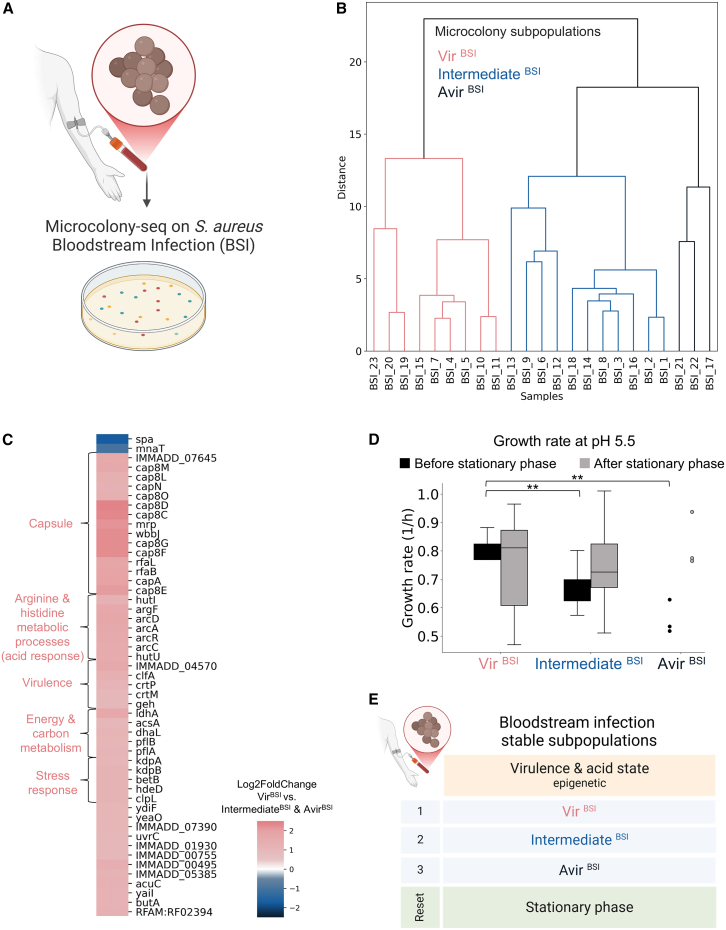
Microcolony-seq reveals antigen expression heterogeneity in a low bacterial load bloodstream *S. aureus* infection (A) Microcolony-seq was performed on blood from a patient with *S. aureus* BSI. 800 μL of blood was plated on tryptic soy agar (TSA), resulting in microcolonies (*n* = 23). (B) Hierarchical clustering of the variable genes in the RNA-seq data identified three subpopulations in the BSI: Vir^BSI^ (*n* = 9), Intermediate^BSI^ (*n* = 11), and Avir^BSI^ (*n* = 3). (C) Heatmap of significantly changed genes (|log_2_fold change| > 1; *p*adj < 0.1) between Vir^BSI^ vs. remainder. Genes upregulated in Vir^BSI^ (pink) include capsule expression, arginine, and histidine pathways (related to acidic environment) and *clfA* adhesion factor. Intermediate^BSI^ and Avir^BSI^ (blue) showed increased *spa* and *mnaT* expression, with SpA enabling immune evasion. (D) The sensitivity of Intermediate^BSI^ and Avir^BSI^ to acid conditions is lost after stationary phase. Growth rate measured in a plate reader in trypticase soy broth (TSB) at acidic pH (5.5). Cultures were inoculated either directly from the original BSI microcolonies or after growth to stationary phase in standard TSB. Acid sensitivity, indicated by a lower growth rate, was observed in the Intermediate^BSI^ (*n* = 11, *p* = 0.005) and Avir^BSI^ (*n* = 3, *p* = 0.002) subpopulations before stationary-phase reset (black) compared with Vir^BSI^ (*n* = 9). However, no significant differences were observed after the stationary phase reset (gray) (*p* = 0.4 and 0.3 when comparing Intermediate^BSI^ and Avir^BSI^, respectively, to Vir^BSI^). Statistical analysis by Student’s t test. (E) Three distinct phenotypic states were detected in the BSI, with acid sensitivity heterogeneity reset at stationary phase. (A) and (E) were created in BioRender. Romm, R. (2025) https://BioRender.com/z60b158. See also Table S6.

Significant efforts have been invested in developing vaccines and antibody therapies against life-threatening *S. aureus* bloodstream infections (BSIs).^88^^,^^89^ Unfortunately, most of these therapies have not succeeded in clinical trials,^90^ likely due to insufficient understanding of *S. aureus* physiology within the host during BSIs. A key challenge is the extremely low bacterial load in the bloodstream, typically below 50 CFU/mL.^91^ Microcolony-seq addresses this limitation by focusing on microcolonies formed from single bacteria, thus requiring only a minimal bacterial count for effective analysis. We applied Microcolony-seq directly to a blood sample from a patient with acute mecillinam-susceptible *S. aureus* (MSSA) bacteremia (Figure 7A). Twenty-three microcolonies appeared after plating ∼800 μL of blood and their WGS revealed that all microcolonies were clonal (Table S6). Strikingly, the transcriptomic analysis of the microcolonies revealed three subpopulations with major differences in the expression of virulence factors, accordingly, named Vir^BSI^, Intermediate^BSI^, and Avir^BSI^ (Figure 7B). Importantly, whereas the Vir^BSI^ displayed the differential expression of 15 capsular genes and additional virulence genes (such as *clfA*, encoding for the clumping factor A ClfA, and toxins), the *spA* virulence factor was expressed only in the Intermediate^BSI^ subpopulation (Figure 7C). The differences in the subpopulations detected in bloodstream bacteremia suggest that therapies targeting factors present in one subpopulation may miss other subpopulations that do not express these factors (Figure 7C; Table S6).

Interestingly, the Vir^BSI^ subpopulation also expressed acid response genes (histidine and arginine metabolism^92^^,^^93^), revealing a link between capsular genes, acid response and adhesion to host cells in *S. aureus* bacteria. The transcriptomic differences in the acid response had functional relevance because the memory of the acid hypersensitivity of the Avir^BSI^ and Intermediate^BSI^ was maintained at the microcolony level and lost after bacteria reached stationary phase (Figures 7D and 7E), revealing the generality of memory loss at stationary phase also for Gram-positive bacteria. This differential memory of acid stress may originate from the biofilm on the patient's heart valve, as biofilm environments are typically acidic.^93^^,^^94^

## Discussion

### Microcolony-seq reveals extensive pathogenic states

The failure of infectious disease therapy has been associated with the ability of bacterial subpopulations to evade the immune system^95^^,^^96^ or antibiotic treatment.^14^^,^^23^^,^^97^ Thus, a deeper understanding of the phenotypic and genetic variability within an infecting strain is essential for developing effective therapeutic solutions. Microcolony-seq provides a methodology for the systematic identification of differentiated pathogenic subpopulations. We demonstrate, using various pathogens, that microcolonies below a certain size can maintain the phenotypic states of the single bacterium from which the microcolony originated. The method takes advantage of the long-term maintenance of the single-bacterium state, not only to analyze its transcriptome but also to combine it with WGS and phenotypic assays on the same subpopulation. This approach revealed phenotypically inherited programs, determined their stability, and allowed the measurement of the fitness advantages associated with each subpopulation.

### Stable memory of host conditions

Bacterial physiology and variability within the human host are often overlooked^98^^,^^99^ due to the difficulty in performing assays on the pathogens directly from patient samples.^100^ Microcolony-seq provides a systematic way to address this issue, requiring only a very low number of bacteria, as demonstrated by analyzing an infected blood sample directly from a patient with less than 30 bacteria per ml. We showed that single bacteria can maintain a long-term memory of their previous host environment, forming differentiated microcolonies on a solid medium. These microcolonies reflected both bacterial physiology and variability within the human host, a key factor in guiding antibiotic therapy.

### Erasure of phenotypic heterogeneity during stationary phase

We identified four types of stably inherited bimodal phenotypes, including Vir^EPEC^/Avir^EPEC^, *fim* ON/OFF, and Vir^UTI^/Avir^UTI^ in *E. coli* and Vir^BSI^/Avir^BSI^ in the Gram-positive *S. aureus*. These phenotypes remained stable across generations, resulting in microcolonies dominated by one phenotype, but were affected by growth to the stationary phase. For example, the bimodal iron utilization phenotype, which correlated with the Vir^UTI^/Avir^UTI^ classification of microcolonies, was observed in microcolonies grown directly from urine and exposed to iron limitation (Figure 6B). However, this pattern was erased when the same bacteria were first grown to stationary phase and then exposed to IL conditions (Figure 6C). The loss of memory observed after growth to stationary phase is probably related to the global transcriptional reprogramming during transition to stationary phase.^101^^,^^102^ Focusing on the Vir^EPEC^/Avir^EPEC^ bimodality (Figure 2E), we showed that the Per proteins contribute to the maintenance of the memory, potentially through positive feedback mechanisms akin to those governing the lactose or arabinose operon in *E. coli*.^4^^,^^28^^,^^71^ The full molecular mechanisms underlying the long-term memory in the other bistable phenotypes remain to be explored.

### Heterogeneity as a strategy for immune evasion and stress adaptation

A feature common to several of the identified phenotypic states is variability in expression of bacterial surface structures, which are essential for host-cell adhesion and virulence (i.e., BFP, T2SS, T3SS, *fim*, flagella, and capsule genes).^56^^,^^103^ Heterogeneity at the single-cell level in *fim* and flagellar expression has recently been identified by par-seqFISH in *Pseudomonas aeruginosa*^104^ and by ProBac-seq in *E. coli*,^40^ underscoring its widespread occurrence. As bacterial surface structures may be easily targeted by the immune system, coexisting populations with or without these structures may provide a bet-hedging strategy, contributing to immune evasion.^6^ Such heterogeneity may also be involved in differential sensitivity to stressful conditions such as antibiotics and antibacterial peptides.^23^^,^^105^^,^^106^

### Revealing fitness advantages of each subpopulation and therapeutic implications

Bacterial strategies such as division of labor and bet-hedging^107^^,^^108^ imply that each subpopulation has some fitness advantages, which contributes to the fitness of the whole population during infection. Although the Vir^EPEC^ subpopulation was shown to gain a clear advantage in host attachment and infection through the T3SS expression,^30^ the Avir^EPEC^ subpopulation exhibited a significant growth advantage under high salt conditions (Figure 2B; Video S1). These observations may indicate a trade-off between membrane robustness and virulence, as seen in *Salmonella*.^109^ Similarly, the Vir^UTI^ subpopulation from the urine infection showed a clear advantage in growth under IL conditions, a critical adaptation in the host environment where immune mechanisms restrict bacterial iron uptake.^87^^,^^110^

The importance of iron metabolism pathways to UTI infections is exploited by therapeutic avenues that take advantage of active iron pathways in UTIs,^111^ for example, vaccination^112^ and antibiotics.^10^^,^^113^^,^^114^ However, the variability of iron utilization pathways that we observed highlights the importance of targeting multiple bacterial subpopulations for effective treatment. This is further emphasized by our analysis of the *S. aureus* subpopulations in the BSI. Proposed therapies like StaphVAX (targeting the capsule),^88^^,^^115^ the monoclonal antibody Tefibazumab (targeting ClfA),^90^^,^^116^ and SA4Ag (targeting ClfA, MntC, and capsule) would presumably eliminate only the Vir^BSI^ subpopulation,^117^ while the 514G3 antibody^118^ (targeting SpA) would presumably eliminate only the Intermediate^BSI^. Therefore, our results may not only explain the failure of such treatments but also point to combinations that may be more effective. Although more patient data are needed to assess whether the identified UPEC and *S. aureus* subpopulations are a common strategy of the pathogens or whether they are patient specific, the ability of Microcolony-seq to unveil and characterize bacterial states within the host makes it a unique tool for drug development and for determining effective drug combinations.

### Limitations of the study

Although Microcolony-seq has revealed substantial heterogeneity in host-associated bacterial populations, it currently relies on relatively low numbers of microcolonies, potentially overlooking rare subpopulations.^14^^,^^39^^,^^40^^,^^41^ Scaling up microcolony sampling with robotic systems could address this limitation. Additionally, Microcolony-seq detects only relatively stable inheritance patterns, potentially missing short-lived variability^119^^,^^120^ that scRNA-seq methods could capture.^35^^,^^36^^,^^37^^,^^38^^,^^39^^,^^40^^,^^41^^,^^42^^,^^43^ Therefore, combining the advantages of Microcolony-seq in identifying genetic differences between subpopulations, which might be incorrectly ascribed to phenotypic variation in other methods, with scRNA-seq,^121^ should optimally provide a comprehensive understanding of bacterial heterogeneity.

### Implications and future prospects

By providing a global landscape of genetic and phenotypic heterogeneity and its phenotypic consequences, Microcolony-seq allows for the systematic identification and characterization of inherited phenotypes without the need for genetic manipulations. Our method should pave the way for detecting many more differentiation strategies used by colony-forming organisms, including fungal pathogens. We have shown that Microcolony-seq enables the detection of phenotypes present in infected patient samples that are lost once re-grown in the lab and therefore inaccessible to standard clinical assays. This knowledge provides a transformative framework for understanding pathogen heterogeneity and paves the way for the development of more precise, effective, and comprehensive therapeutic strategies.

## Resource availability

### Lead contact

Further information and requests for resources and reagents should be directed to, and will be fulfilled by, the lead contact, Nathalie Q. Balaban (nathalie.balaban@mail.huji.ac.il).

### Materials availability

The strains generated and isolated in this study are available upon request.

### Data and code availability

RNA-seq data of Microcolony-seq experiments generated in this study for EPEC and for the UTI sample are available at ArrayExpress, Database: E-MTAB-13486 and E-MTAB-13892, respectively. The RNA-seq data for the BSI sample are available at Biostudies, Database: S-BSST1842. WGS of EPEC anaerobic microcolonies, UTI microcolonies, and BSI microcolonies, Database: E-MTAB-13428, E-MTAB-13941, and S-BSST1859, respectively. The PacBio sequencing file for UTI_30 sequencing is available, Database: E-MTAB-13944, and the Fasta file of the UPEC assembled genome is available at GenBank: SAMN41600000. The Fasta file of the *S. aureus* assembled genome is available at GenBank: SAMN46777821. Code for determining variable genes in RNA-seq data is available at https://doi.org/10.5281/zenodo.16275975. Any additional information required to reanalyze the data reported in this paper is available from the lead contact upon request.

## Acknowledgments

We thank H. Margalit, A. Rotem, H. Rappeport, Y. Altuvia, and N. Livne for helpful advice. We thank S. Pearl Mizrahi, I. Levin-Reisman, and all the Balaban lab members for fruitful discussions. We thank L. Argaman for help with sequencing protocols and A. Bar for help with the genome browser. We thank Y. Kaplan and K. Cohen for experimental support and E. Romm for graphical help. We thank A. Nasereddin from the Genomics Applications Laboratory, Core Research Facility, Faculty of Medicine, and A. Turjeman from the Center for Genomic Technologies of the Hebrew University of Jerusalem for help with sequencing experiments. We thank Y. Nevo, from the Info-CORE, Bioinformatics Unit of the I-CORE Computation Center at the Hadassah Medical School, for helpful advice. We thank M. Love for the support with DESeq2, J. Barrick for the support with *breseq*, and R. Chaudhuri for the help with gene annotations. This study was supported by the European Research Council Advanced Grant (101054653), The Minerva Center for Stochastic Decision Making in Microorganism, and the Israel Science Foundation (597/20). R.F.-R. was partially supported by the Emily Erskine Endowment Fund for post-doctoral research fellows. I. Rosenshine was supported by grants from the European Research Council (810186) and the Israel Science Foundation (743/18).

## Author contributions

Conceptualization, N.Q.B., R.F.-R., and I. Rosenshine; methodology, N.Q.B., R.F.-R., and I. Rosenshine; investigation, R.F.-R., O.G., N.K.-N., N.Y., L.A., and I. Ronin; software, R.F.-R.; data curation, R.F.-R. and N.Y.; writing – original draft, N.Q.B. and R.F.-R.; writing – review and editing, N.Q.B., R.F.-R., I. Ronin, O.G., N.K.-N., N.Y., L.A., M.B.-M., and I. Rosenshine; funding acquisition, N.Q.B.; resources, N.Q.B., R.F.-R., I. Ronin, O.G., N.K.-N., M.B.-M., and I. Rosenshine; and supervision, N.Q.B.

## Declaration of interests

The authors declare no competing interests.

## STAR★Methods

### Key resources table

**Table:** 

REAGENT or RESOURCE	SOURCE	IDENTIFIER
**Antibodies**		

anti-EPEC	Prof. Ilan Rosenshine	N/A
anti-Rabbit-alexa488	Cell Signaling	Cat# 4412; RRID:AB_1904025
phalloidin rhodamine	Sigma-Aldrich	Cat# P1951; RRID:AB_2315148
anti-FLAG	Sigma-Aldrich	Cat# F1804; RRID:AB_262044
anti-Mouse-HRP	Cell Signaling	Cat# 7076; RRID:AB_330924
Anti-H6	Statens Serum Institut	N/A

**Bacterial and virus strains**		

E2348/69, EPEC wild type (strain O127:H6)	Ronin et al.^30^	N/A
EPEC *ΔperA*, perABC-GFP	Ronin et al.^30^	N/A
EPEC pZS^∗^11-GFP	Ronin et al.^30^	N/A
EPEC pilW-FLAGx3:kan	This study (primers 7633+7636)	NY12040
EPEC Δ*bfpA* pilW-FLAGx3:kan	This study (primers 7633+7636)	NY12062
EPEC Δ*pilW*:kan	This study (primers 7632+7633)	NY12038
EPEC / pZE21-pilW (pNY12057)	This study (primers 7640+7641, 6466+7105)	NY12060
EPEC / p15A-gfp (pNY10020)	This study (primers 936+6313, 5651+6312)	NY11113
EPEC Δ*pilW*:FRT / p15A-gfp	This study	NY12134
EPEC WT perB-FLAGx3	This study (primers 5861+5862)	LA9372
EPEC Δ*pilW*:FRT / pZE21-pilW (pNY12057) / p15A-gfp	This study	NY12136

**Biological samples**		

Urine of a patient with a UPEC urinary tract infection	The urine sample was obtained with written informed consent of the participant.	N/A
Infant stool	Stool sample was obtained with written consent of parents according to ethics committee guidelines	N/A
Blood sample with *S. aureus* bloodstream infection	The blood was collected from a patient with a bloodstream infection. Helsinki: 0031-22-SZMC	N/A

**Chemicals, peptides, and recombinant proteins**		

Oxyrase Enzyme System for Agar	Sigma-Aldrich	Cat#SAE0058
2,2′-Dipyridyl	Sigma-Aldrich	Cat#D216305
E-test Trimethoprim/sulfamethoxazole (1/19) (TS)	bioMerieux	Cat#412481
E-test Streptomycin SM 1024	bioMerieux	Cat#526800
Sodium butyrate	Sigma-Aldrich	Cat#303410
DMEM, high glucose, HEPES, no phenol red	Thermo Fischer Scientific	Cat#21063029
RNase A/T1 mix	Thermo Fischer Scientific	Cat#EN0551
T4 Polynucleotide Kinase	New England Biolabs	Cat#M0236L
T4 RNA ligase 1, High Concentration	New England Biolabs	Cat#M0437M
RNAClean XP	Beckman Coulter	Cat#A63987
AMPure XP	Beckman Coulter	Cat#A63881
Recombinant RNase inhibitor (RRI)	Takara	Cat#2313A
TURBO DNase	Thermo Fischer Scientific	Cat#AM2238
FastAP	Thermo Fischer Scientific	Cat#EF0654
RLT buffer	Qiagen	Cat#79216
Glycoblue	Thermo Fischer Scientific	Cat#AM9516
TriReagent	Sigma-Aldrich	Cat#T9424
Dynabeads® MyOneTM Streptavidin C1	Thermo Fischer Scientific	Cat#65001
EDTA (0.5 M), pH 8.0, RNase-free	Thermo Fischer Scientific	Cat#AM9260G
Sodium Acetate (3 M), pH 5.5, RNase-free	Thermo Fischer Scientific	Cat#AM9740
Tris (1 M), pH 7.0, RNase-free	Thermo Fischer Scientific	Cat#AM9850G
Tris (1 M), pH 8.0, RNase-free	Thermo Fischer Scientific	Cat#AM9855G
SSC (20X), RNase-free	Thermo Fischer Scientific	Cat#AM9770
NaCl (5 M), RNase-free	Thermo Fischer Scientific	Cat#AM9760G
IGEPAL	Sigma-Aldrich	Cat#I8896
Glass beads, 0.1-mm diameter	Biospec	Cat#11079101
TriReagent LS	Sigma-Aldrich	Cat#T3934
Sodium Phosphate Buffer, 0.2M, DEPC Water, pH 8.0	Tivan Biotech	Cat#SP02D80
Fetal calf serum (FCS)	Gibco	Cat#A5256801
Penicillin-Streptomycin (pen-strep) (10,000 U/mL)	Gibco	Cat#15140122
DMEM (for infection experiments)	Sigma-Aldrich	Cat#D5796

**Critical commercial assays**		

DNeasy Blood & Tissue Kit	Qiagen	Cat#69504
SuperScript III first strand kit	Invitrogen	Cat#18080-051
HIFI HotStart RM	Kapa Biosystems	Cat#KK2601
RNA clean and Concentrator™-5 kit	Zymo Research	Cat#R1016

**Deposited data**		

Whole genome sequencing of EPEC microcolonies from anaerobic conditions	This work	E-MTAB-13428
RNA-seq files of Virulent and Avirulent EPEC microcolonies from aerobic, butyrate, infant stool metabolites and anaerobic conditions	This work	E-MTAB-13486
RNA-seq files of Microcolony-seq applied on a clinical urinary tract infection	This work	E-MTAB-13892
Whole genome sequencing of urinary tract infection (UTI) microcolonies used for Microcolony-seq	This work	E-MTAB-13941
Pacbio sequencing of bacteria regrown from one colony from a urine of a patient with a urinary tract infection	This work	E-MTAB-13944
Fasta file of the UTI bacteria assembly by Pacbio and PE150 sequencing	This work	Accession number JBEAAH000000000; BioSample SAMN41600000
RNA-seq fastq files of Microcolony-seq applied on a bloodstream infection	This work	S-BSST1842
Whole genome sequencing (WGS) of *S. aureus* microcolonies from a bloodstream infection	This work	S-BSST1859
Fasta file of the *S. aureus* genome from the bloodstream	This work	BioSample accession: SAMN46777821

**Experimental models: Cell lines**		

HeLa cells	ATCC	CCL-2

**Oligonucleotides**		

Library preparation oligos	Table S1	N/A
rRNA depletion oligos	Table S1	N/A
EPEC strain construction oligos	Table S1	N/A
PCR oligos	Table S1	N/A

**Software and algorithms**		

Breseq	Deatherage and Barrick^122^	https://github.com/ba rricklab/breseq
Unicycler	Wick et al.^123^	https://github.com/rr wick/Unicycler
Prokka	Seemann^124^	https://github.com/ts eemann/prokka
ScanLag	Levin-Reisman et al.^53^	https://github.com/of erfrid/NQBMatlab/tre e/V16
DESeq2	Love et al.^54^	R package. In R: BiocManager::install( "DESeq2")
variable_genes.py	This work	Python function in https://github.com/R aya-FaigenbaumRomm/Microcolonyseq/; DOI: https://doi.org/10.5281/zenodo.16275975

### Experimental model and study participant details

#### Bacterial Strains

Bacterial strains used in this work are detailed in the key resources table.

For EPEC experiments: Frozen stock was streaked on LB Broth (Lennox) medium (Sigma-Aldrich) and incubated overnight at 37°C. Colonies were picked and grown overnight in 2mL LB. Culture was divided in 50μL aliquots with glycerol (15% final concentration) and kept at -80°C.

Host-mimicking conditions: EPEC wild type (strain E2348/69, O127:H6) bacteria from frozen aliquot (diluted 1:40) were grown overnight in LB with streptomycin (50 μg/ml) at 37°C without shaking, diluted 1:40 into DMEM-HEPES (Gibco, Israel) medium and incubated for 3h at 37°C without shaking to exponential phase (optical density (OD) ∼ 0.5). For ScanLag analysis, bacteria were plated on LB plate with streptomycin (50 μg/ml), incubated at 32°C for 15h (for aerobic LB) or for 12h (for aerobic butyrate and infant stool metabolites conditions) or at 37°C (for anaerobic condition) for 12 h. Colonies of two sizes (Vir^EPEC^ & Avir^EPEC^) were sampled.

Aerobic LB agar plates containing infant stool metabolites: stool of a 10-month-old infant was resuspended in sterile NaCl 0.9% solution and filtered with PVDF membrane Millex-GV Low protein binding Durapore membrane 0.22μm to obtain the supernatant containing the stool’s metabolites. LB agar was autoclaved and cooled to 48^°^C, infant stool metabolites were added. Infant stool samples were obtained with written consent of the parents.

Aerobic LB agar plates containing butyrate: LB agar was autoclaved and cooled to 48°C, butyrate at a final concentration of 50mM (resembling the concentration in the small intestine) was added.

Anaerobic LB plates: LB agar was autoclaved and cooled to 48^°^C. Oxyrase for Agar (Sigma-Aldrich, SAE0058), was thawed, brought to room temperature and added to the LB (8 mL Oxyrase for 200 mL LB, according to manufacturer instructions). After plating bacteria on the anaerobic plates, they were sealed with parafilm before incubation.

#### Human Subjects

Samples were collected with consent (Helsinki: 0031-22-SZMC). Urine was collected from a 56-year-old, previously healthy female. Blood was collected from a 12-year-old, previously healthy Caucasian boy. Infant stool was collected from a 10-month-old healthy male infant.

### Method details

#### Microcolony-seq methodology

##### Experimental pipeline of Microcolony-seq

*Microcolony isolation*. Bacteria cultured in liquid media were plated on agar plates. Thorough resuspension is required before plating to ensure that microcolonies are founded by single cells. To pick microcolonies as early as possible for the Microcolony-seq experiment, it was essential to determine their appearance time on the agar plates. This could be achieved manually by inspecting the plates at short intervals, or by using the automated ScanLag technique^53^^,^^83^ (Figures S1A–S1G). Briefly, the ScanLag method consists of using regular agar plates placed on office scanners flatbed located in a temperature-controlled room or in an incubator. The ScanLag software controls the scanners and acquires images every 15 minutes. Subsequently, a Matlab code extracts the appearance time and growth rate of each detected microcolony from the acquired images. This facilitated picking microcolonies as soon as detectable from the agar plates using a sterile plastic stick using the round end. Each microcolony was resuspended in 100 μl ice-cold TE buffer (10 mM Tris-HCl pH 7.5, 1 mM EDTA). Each microcolony consisted of ∼10^6^ bacteria.

*Microcolony material storage*. 10 μl of each microcolony resuspension was diluted in 500 μl LB with 15% glycerol in a separate tube and stored at −80°C for further analysis. Aliquots of the stored material were prepared to prevent thawing and re-freezing of bacterial samples, which may lead to the loss of phenotypic stability and identity.

*Microcolony RNA-seq*. The remaining 90 μl microcolony resuspension was exposed to lysosyme (0.9 mg/ml, Sigma-Aldrich). The samples were immediately frozen in liquid nitrogen and stored at -80°C. RNA was extracted with 1 mL TriReagent (Sigma-Aldrich) per sample, as described in Faigenbaum-Romm et al.^125^ In detail, frozen samples were subjected to two freeze-thaw cycles by incubating at 37°C and then snap-freezing in liquid nitrogen. After the second cycle, samples were thoroughly mixed with 1 mL of TriReagent (pre-warmed to room temperature) and incubated for 5 minutes at room temperature to allow complete lysis. Following lysis, 200 μL of chloroform was added to each sample. Tubes were inverted gently for 15 seconds to mix and then left to stand at room temperature for 10 minutes. Samples were centrifuged at 17,000g for 10 minutes at 4°C, and the upper phase was carefully transferred to new Eppendorf tubes. To precipitate RNA, 500 μL of isopropanol was added to each tube. To aid visualization of the RNA pellet, especially given the expected low yield from very small microcolonies, 1.5 μL of Glycoblue (Thermo Fischer Scientific) was added to each sample. The contents were mixed by inversion and incubated over-night at -20°C. Samples were centrifuged again at 17,000g for 15 minutes at 4°C, and the resulting supernatant was discarded. RNA pellets were washed twice with 1 mL of freshly prepared 75% ethanol, each wash followed by centrifugation at 17,000g for 5 minutes at 4°C. After the final wash, pellets were air-dried at room temperature for 15 minutes, then resuspended in 20 μL of nuclease-free water. RNA samples were stored at -20°C until use. RNA quality was assessed by Nanodrop spectrophotometer and by Bioanalyzer using the Agilent RNA 6000 Pico Kit (5067-1513).

rRNA depletion was done with the DIY rRNA depletion method,^126^ using the rRNA sequence probe set designed for *E. coli* K-12 and for Gram-negative bacteria for the EPEC and UPEC libraries and the probe set designed for *S. aureus* and for Gram-positive bacteria for the *S. aureus* libraries (Table S1, Oligo sheet). RNA-seq libraries were constructed based on the RNAtag-Seq protocol^127^ with several modifications.^128^ The rRNA depletion step was done before the fragmentation step in the RNAtag-Seq protocol. Then, the first ligation was carried out and the rest of the RNAtag-Seq protocol was followed. The libraries were single-end sequenced using the Nextseq500 Sequencer (Illumina) (for oligonucleotides used for the RNA-seq libraries see Table S1).

##### Computational pipeline of Microcolony-seq on EPEC microcolonies

Analysis of RNA-seq data was done using the code *process_nextseq_run.py* (https://github.com/asafpr/RNAseq_scripts/blob/master/process_nextseq_run.py). The code uses *FastQC* version v0.11.8^129^ for sequence quality control before and after adaptor removal, *cutadapt*^130^ version 3.5 with Python 3.7.12 for adaptor removal, *SAMtools*^131^ version 1.9 for indexing, *bwa*^132^ version 0.7.17-r1188 for mapping. Reads were mapped to EPEC E2348/69 genome version 19, including chromosome NC_011601.1 and three plasmids NC_011602.1, NC_011603.1, and EU580135, with additional annotations defined in Pearl Mizrahi et al.^51^ Mapping information for the RNA-seq can be found in Table S1. After mapping, the *process_nextseq_run.py* intersects the mapped reads with a pre-defined gene annotation file and outputs the number of reads that were mapped to each gene. These count files are used as an input for the DESeq2^54^ analysis (version 1.30.1) which was used for normalization of sequencing raw-counts. Genes with lower mean expression of 10 were excluded from the analysis. DESeq2 was used with default parameters.

*Identification of variable genes in biological replicates*. Following normalization, the dispersion, $d_{i}$, of gene i, similarly to the one calculated in DESeq2,^54^ was calculated by:

(Equation 1) $$d_{i}=\frac{v_{i}-m_{i}}{m_{i}^{2}}$$

Where $v_{i}$ is the variance and $m_{i}$ is the mean expression, both calculated for gene i across all genes and all samples (microcolonies). In Figure 1B is plotted a typical example of $d_{i}$ vs. $m_{i}$. Our goal was to identify the most variable genes, i.e. with a higher dispersion than expected from the typical trend of the dispersion versus the mean. For this purpose, we divided the genes according to bins of mean expression. Genes with a mean expression level below 10 were excluded from the analysis. For each gene i, we calculated the log_10_ of its mean expression ($m_{i}$) and multiplied this value by 100. The bins were then defined as follows: starting from 100 to 275 in increments of 25, with the final bin containing all genes with a log_10_($m_{i}$) × 100 value greater than 275. Each gene was assigned to a bin based on this value, and the mean dispersion for each bin was calculated. Genes whose dispersion values were more than two standard deviations above the mean for their respective bin were classified as dispersion outliers, i.e., were identified as variable genes. The genes defined as variable genes were used for the PCA of the sampled microcolonies, excluding the genes found to be variable also in the technical replicates genes (see below). The code for identification of variable genes is available at: https://github.com/Raya-Faigenbaum-Romm/Microcolony-seq/ (https://doi.org/10.5281/zenodo.16275975).

The subpopulations of microcolonies defined by the PCA plot were then used for the differential expression analysis across all genes in the genome (DESeq2^54^). DESeq2 was used with default parameters. Genes with padj < 0.1 and with |log2FoldChange|>1 values were considered as statistically significantly changed between the two subpopulations, unless indicated otherwise.

*Identifying the variable genes in technical replicates*. To avoid including RNAs which are inherently prone to variability due to technical issues, and to improve the robustness of the methodology, we performed the analysis of RNA-seq on technical replicates. Four Vir^EPEC^ and four Avir^EPEC^ microcolonies from the aerobic condition were picked using a sterile stick from the same agar plate as described above, mixed together and resuspended in 400 μl ice-cold TE buffer in a single Eppendorf tube. Each 100 μl were then divided into separate Eppendorf tubes for processing with Microcolony-seq, as described above. Variable genes were calculated separately for the technical replicates, and any gene determined as variable gene in the technical samples was excluded from the analysis of the biological samples (Figure S1H). This step helped to account for technical variability, such as RNA extraction, library preparation or sequencing biases resulting from variations in RNA structure or length. The PCA of the aerobic condition including Vir^EPEC^ and Avir^EPEC^ biological and technical microcolonies can be found in Figure S1I.

#### Microcolony-seq applied on a UTI sample

Urine was obtained from a patient with a urinary tract infection untreated with antibiotics and frozen shortly after extraction in 15% glycerol at -80°C. A sample from the frozen urine was serially diluted 1:10^4^, thoroughly resuspended, plated on LB agar, and incubated at 37°C for 8h. The ScanLag setup was used to measure the distribution of microcolony appearance times.^53^ Microcolonies were visible after 8 hours of incubation. A unimodal distribution of appearance time was observed (Figures S6A and S6B). Twenty microcolonies were picked and subjected to the Microcolony-seq pipeline. Four additional microcolonies were mixed and split into four Eppendorf tubes for the technical replicates. Mapping information for the RNA-seq can be found in Table S1.

When the UTI microcolonies were clustered based on the variable genes, excluding those that lost the antibiotic resistance cluster (Figure 5B), one microcolony (indicated in grey in the PCA plot in Figure 5D) could not be assigned to any subpopulation and was thus excluded from the analysis. Including this microcolony in either of the two subpopulations (Vir^UTI^ or Avir^UTI^) did not affect the identified gene groups in the comparison between Vir^UTI^ and Avir^UTI^ subpopulations. The PCA including of studied UTI microcolonies as well as the technical replicates can be found in Figure S6E.

#### Microcolony-seq applied on blood from a bloodstream infection

Blood was collected from a patient with an *S. aureus* bloodstream infection. The patient was diagnosed with bacteremia, endocarditis and osteomyelitis. A transthoracic echo demonstrated a vegetation on the tricuspid valve. Prior to sample collection, the patient was treated with vancomycin and ceftriaxone. Whole blood was frozen shortly after collection in 15% glycerol at -80°C. A volume of 800μL of the blood sample was plated on Tryptic Soy Agar (TSA) plates and incubated at 37°C. After 11h of incubation, 23 microcolonies were observed and subsequently analyzed using the Microcolony-seq pipeline. Due to the low bacterial load in the bloodstream infection, no technical replicates were included in the BSI Microcolony-seq experiment. Instead, all observed microcolonies were utilized as biological replicates for the analysis. For RNA extraction, frozen microcolonies were resuspended in 300 μl of Salts solution (50mM Sodium phosphate, 300mM NaCl) supplemented with 10 μl of Recombinant RNase Inhibitor (RRI) and 25 μl of IGEPAL per 10 ml of Salts solution. Each sample was transferred to an Eppendorf tube containing 200μl of 0.1-mm glass beads (Biospec, cat. no. 11079101). Samples were subjected to ten cycles of vigorous vortexing (30 seconds) followed by incubation on ice (30 seconds). Samples were centrifuged at 17,000 g for 10 minutes, and the supernatant was transferred to a new Eppendorf tube. RNA was extracted by adding 900 μl of pre-warmed TriReagent LS (Sigma-Aldrich, cat. no. T3934), for processing liquid samples following the same protocol as described above for RNA extraction using TriReagent.

#### Whole genome sequencing and analysis

Bacteria from the original microcolonies analyzed by Microcolony-seq and kept frozen at -80°C were regrown in liquid LB at 37°C with shaking (200 rpm) for 5 hours to OD 0.1. Genomic DNA was extracted from 1 mL of bacteria using the DNeasy Blood & Tissue Kit (Qiagen). For genomic DNA extraction from *S. aureus*, following growth, bacteria were centrifuged at 4,500 g for 10 minutes and resuspended in 300μl of ALT buffer (from the DNeasy Blood & Tissue Kit). Each sample was transferred to an Eppendorf tube containing 300μl of 0.1-mm glass beads (Biospec). Samples were subjected to five cycles of vigorous vortexing (30 seconds) followed by incubation on ice (30 seconds). Samples were centrifuged at 17,000 g for 10 minutes, and the supernatant was transferred to a new Eppendorf. DNA purification then proceeded according to the DNeasy Blood & Tissue Kit was followed.

##### WGS of EPEC microcolonies

As the E2348/69 strain genome is known, only short reads were used. Library preparation and sequencing was carried out by the Genomics Applications Laboratory, Core Research Facility, Faculty of Medicine, The Hebrew University of Jerusalem, Jerusalem, Israel. DNA libraries for sequencing were prepared using the kit (ILMN DNA LP (M) Tagmentation (24 Samples, IPB) 20060060) (Illumina, Inc., USA) according to manufacturer’s instructions. At least 100× sequencing depth for each nucleotide in each sample was targeted. Samples were sequenced with the Nextseq500 machine using the 150-cycle mid output kit (Illumina, Inc., USA) from the forward read direction.

Sequencing reads were analyzed by removing adaptors using cutadapt^130^ (Nextera_forward adaptors). Sequencing quality was tested by FastQC.^129^ *breseq*^122^ with default parameters was used for identification of mutations and junctions. Data is available in Table S3.

##### WGS of UTI microcolonies

In order to perform de-novo assembly of the genome of the clinical strain, short- and long-read sequencing were combined. Library preparation and sequencing was carried out by BGI company, China. For short-reads, samples were sequenced with the DNBseq G400 machine using the 150-cycles paired-end with 350 bp insert size. At least 150× sequencing depth for each nucleotide in each sample was targeted.

PacBio long-reads library preparation and sequencing was carried-out by BGI on one of the technical samples (UTI_30). SMRTbell prep kit 3.0 was used for library preparation and the SMRTbell library was sequenced in a PacBio Revio machine. The PacBio data along with the paired-end data for the same sample were assembled using Unicycler.^123^ Microcolony #UTI_17 (containing the siderophores and the antibiotic resistance gene clusters) was mapped to the assembled genome using *breseq*.^122^ The unmapped reads were assembled using Unicycler.^123^ Prokka^124^ was used to annotate the assembled contigs from both Unicycler runs. The assembled genome was used for the whole-genome analysis of all microcolonies by *breseq* and for the RNA-seq analysis. The mapping percentage for the WGS of UTI microcolonies was above 99.7% for all microcolonies. BioSample accession: SAMN41600000. Data is available in Table S5.

##### WGS of BSI microcolonies

To perform de-novo assembly of the genome of the clinical strain, short- and long-read sequencing were combined. Library preparation and sequencing was performed by Plasmidsaurus company, USA. Short reads were obtained by paired-end Illumina sequencing and long-read sequencing of microcolony #22 (BSI 22) was done using Oxford Nanopore Technology. Genome de-novo assembly and annotation was performed by Plasmidsaurus. The assembled genome was used for the whole genome analysis of all microcolonies by *breseq* and for the RNA-seq analysis. BioSample accession: SAMN46777821. Data is available in Table S6.

#### Phylogenetic tree

To visualize the genetic differences identified in the whole-genome sequencing of the microcolonies from the UTI sample, a phylogenetic tree was generated with gdtools COMPARE from the *breseq* package^122^ using phylip, the phylogeny Inference Package.^133^ The phylogenetic tree was visualized using Newick Viewer^134^ and can be found in Figure S6F.

#### Soft agar motility assay

Bacteria were inoculated with a plastic stick in the middle of fresh aerobic soft agar plates (Tryptone 1 gr, NaCl 0.5 gr, Agar 0.25 gr in 100mL Double-distilled water) at 37°C, the plates were imaged every 30 min using automated imaging (ScanLag).^53^ The motility area was quantified using ImageJ software.^82^

#### PCR

To validate *lrhA* mutations identified in the whole genome sequencing, primers lrhA-F and lrhA-R were used in the PCR experiments.

To examine the direction of the *fim* operon, PCR for amplification of two fragments was carried out for the *fim* ON direction using primers FimS and FimE-Rev and for the *fim* OFF direction using primers FimA-Rev and FimS. To examine the possible reset on the *fim* ON state after stationary phase, EPEC microcolonies #1 and #2 from the anaerobic condition kept at -80°C were grown for 20h in standard LB and diluted 1:100 in LB and grown for 3h at 37°C with shaking. To examine the possible reset on the *fim* OFF state, frozen EPEC microcolonies #3 and #4 were grown for 40h in DMEM at 37°C without shaking, diluted 1:100 and grown for 2h in DMEM at 37°C without shaking. A comparable number of bacteria of the frozen and after re-growth was used for the PCR.

Primer sequences can be found in Table S1 in Oligo sheet.

#### Microscopy

Microscopy was performed using a NIKON Ti2 inverted microscope system with TANGO motorized XY stage (MARZHAUSER). The microscope was placed in a cage incubator box at 37°C (OKOLAB). Image acquisitions were done using Micro-Manager^135^ to control the microscope, stage and camera. Several different locations were monitored in parallel on the same sample. Images were acquired using a 100x /1.45NA oil objective and sCMOS camera (ORCA FLASH 4.0V3-DIGITAL, Hamamatsu).

Fluorescence images were acquired with minimal excitation to minimize bleaching (Spectra X (Lumencor), EX filter 500/24 EM 542/27 (Semrock)). No difference was noticed in the growth of cells (on agar pads) exposed or unexposed to fluorescence illumination.

For high salt conditions, agar pads cut out from sterile LB agar plates with NaCl concentrations of 0.05M (standard LB) or 0.7M (high salt) were placed on slides with 2μL of bacteria. Slides were sealed with a plastic wrap. The EPEC pZS^∗^11-GFP strain was included as a control to confirm the ability of bacteria to produce GFP under high salt conditions.

For observation of motility at the single-cell level, agar pools were prepared by pouring LB agar on top of silicon wafer patterned with microwells (Figure S5D). The pattern was done using photolithography with SU-8 photoresist (MicroChemCorp, MA), as previously described^136^ resulting in a pattern of microwells (100 μm depth and 450 μm diameter). A volume of ∼2μL of bacteria was placed on a slide and covered by the agar pools pad.

#### Growth of UPEC under iron-limiting conditions

Iron-limited LB plates (IL) were prepared by adding 2,2′-Dipyridyl (DPY, Sigma, diluted in ethanol) to LB agar at a final concentration of 300 μM. This was done after autoclaving the agar and allowing it to cool to 48°C. Bacteria from the original microcolonies (n = 4 for Avir^UTI^ and n = 4 for Vir^UTI^) kept frozen at −80°C were streaked on standard LB and on IL plates and incubated at 37°C. To examine regrowth from stationary phase colonies on IL plates, we first allowed colonies (n = 4 for Avir^UTI^ and n = 4 for Vir^UTI^) to grow on standard LB plates during a 24-hour incubation. These colonies were then re-plated on IL plates. Due to batch variation in the effectiveness of iron limitation, quantitative results were compared only between plates of the same batch.

#### Growth of *S. aureus* under acidic conditions

##### Before stationary phase growth

*S. aureus* bacteria derived from the original microcolonies used in the BSI Microcolony-seq experiment were grown in either standard Tryptic Soy Broth (TSB) or TSB adjusted to pH 5.5 with HCl. The experiment was performed in 96-well plates with orbital shaking (2.5 mm amplitude) using a plate reader (Infinite M200 Pro, Tecan) at 37°C, with a total volume of 200 μL per sample.

##### After stationary phase growth

Following 20 hours of growth in standard TSB (without acid stress), *S. aureus* cultures were diluted 1:200 into either fresh standard TSB or fresh acidic TSB (pH 5.5) and incubated under similar conditions for an additional 24 hours. Growth rates for each microcolony were quantified using a custom Python script.

All 23 microcolonies were tested: 9, 11 and 3 from the Vir^BSI^, Intermediate^BSI^ and Avir^BSI^, respectively. Statistical significance among the BSI subpopulations before and after stationary phase was assessed using the Mann–Whitney U test in R.

#### E-testing of UPEC bacteria

Frozen bacteria obtained from microcolonies used in the Microcolony-seq analysis of the UTI clinical sample were diluted 1:200 and cultured in 1mL LB at 37°C with shaking in a 24-well plate in a plate reader until the bacterial culture reached the OD of 0.1 (∼8h). Subsequently, the bacteria were plated on LB agar and subjected to E-tests (bioMérieux SA). The plates were incubated at 37°C for 18 hours and imaged with an office scanner.

#### Western blot

EPEC bacteria were grown overnight standing in LB+antibiotics, diluted in DMEM 1:50 and incubated in 50mL tubes at 37°C, 5% CO_2_ for several hours. At ∼0.5 OD_600_ for logarithmic phase, or ∼1.5 OD_600_ for stationary phase, bacterial cells were collected and treated with sample buffer. Samples were boiled for 10 min, centrifuged, and run in a protein gel. After transfer, membranes were treated with blocking solution (2% BSA in TBS) overnight at 4°C. Membranes were then treated with primary antibody solution in TBS-tween (TBST) for 1 hour at room temperature, washed twice with TBST and treated with secondary antibody for 1 hour at room temperature. Blots were developed with ClarityMax™ (biorad #1705062) and ChemiDoc™ imager (biorad).

#### Bacterial attachment – immunostaining & fluorescent microscopy (EPEC)

For experiments included in Figure 1F: HeLa cells were grown overnight in DMEM supplemented with 10% fetal calf serum (FCS) (Gibco) and with 1% pen-strep (Gibco), in a 24 well plate, containing coverslips, 8×10^4^ cells per well. EPEC bacteria were grown overnight without shaking in LB+antibiotics. Wells were washed twice with PBSx1, then infected with bacteria in fresh DMEM at MOI of 1:100, and incubated at 37°C, 5% CO2 for 3.5 hours. After infection, coverslips were washed twice and fixed with 3.7% formaldehyde for 10 min. After fixation, coverslips were washed twice and treated with 0.25% triton x-100 in TBSx1 for 10 min. To stain EPEC cells, coverslips were treated with anti-EPEC antibody solution (1:1000) for 1 hour. Coverslips were washed 3 times and treated with anti-rabbit-alexa-488 antibody (1:1000) + phalloidin rhodamine (1:700) solution for 1 hour. Then coverslips were washed 3 times and mounted on microscope slides with mounting media (Immu-MountTM, 9990402, epredia) and left to dry overnight. Images were taken using Nikon ECLIPSE Ti2 microscope & PrimeBSITM camera (Teledyne), and analysed with NIS-Elements software.

For experiments included in Figure 4C: HeLa cells were grown similarly as described above. Bacteria from Vir^EPEC^ Hyper-flagellated and Non-flagellated subpopulations were added to wells containing the HeLa cells directly from the frozen microcolonies from the anaerobic EPEC Microcolony-seq experiment (20μL for each). HeLa cells were infected for 3h. Coverslips were washed and fixed as described above. Flagella was stained using anti H6 antibody (1:200) and the secondary antibody was anti-rabbit-alexa-488 antibody (1:1000) + phalloidin rhodamine (1:700).

#### Bacterial attachment – flow cytometry

HeLa cells were grown overnight in DMEM supplemented with 10% FCS and with 1% pen-strep, in a 6 well plate, 3.5×10^5^ cells per well. EPEC bacteria carrying p15A-gfp plasmid with an IPTG (Isopropyl β-D-1-thiogalactopyranoside) inducible Ptac promoter (strains NY11113, NY12134 and NY12136) were grown overnight without shaking in LB+Amp. HeLa cells were washed twice with PBSx1, then infected with bacteria in fresh DMEM in MOI of 1:100, induced with 0.2mM IPTG and incubated at 37°C, 5% CO_2_ for 2.5 hours. After infection, the plate was washed 3 times, and HeLa cells were scraped from the well surface into 400μl PBSx1, then fixed in 3.7% formaldehyde in Eppendorf tubes. All samples were measured with CytoFLEX 5 instrument & CytExpert software for levels of GFP signal per cell. Data were analyzed and visualized using floreada.io website (https://floreada.io).

### Quantification and statistical analysis

To compare gene expression levels between the different subpopulations, differential expression analysis was performed using the DESeq2 package in R^54^ and detailed analysis was described in relevant method sections.

Statistical details, including numbers of microcolonies or samples and types of statistical analyses can be found in figure legends and results sections.

#### Identification of putative virulence genes

To identify unannotated putative virulence genes in EPEC genome, we compared the wig files of the sequencing files between the Vir^EPEC^ and Avir^EPEC^ microcolonies. We looked for continuous regions larger than 100 nucleotides in the EPEC genome with at least two-fold higher expression level between the two types of microcolonies. After retrieving these regions, we looked for an open reading frame using ORFinder (NCBI). Regions with an open reading frame were included in the gff file of the annotated genes in EPEC to obtain the counts for these regions. Differential expression analysis using DESeq2^54^ was carried out to determine which of these regions are statistically significantly increased between Vir^EPEC^ and Avir^EPEC^ microcolonies.

## Supplemental information

Table S1. RNA-seq mapping information and oligos, related to Figures 1, 3, 4, and 5 and STAR Methods RNA-seq reads sheet: Included are the numbers of total reads, processed and mapped reads for all Microcolony-seq sequencing libraries included in this study. Strict mapping included only reads that were mapped uniquely to one genomic location. Oligos sheet: List of oligonucleotides used in this study.

Table S2. Differential expression analysis between Vir^EPEC^ and Avir^EPEC^ microcolonies and putative virulence genes in EPEC, related to Figures 1 and 2 Virulent vs Avirulent sheet: Included are genes that were significantly changed between Vir^EPEC^ and Avir^EPEC^ microcolonies (*padj* < 0.1) in one of the growth conditions. For each gene and for each growth condition, the mean expression between all microcolonies (basemean), the log2FoldChange, and *padj* calculated by DESeq2^54^ were indicated, and columns were color coded by condition: green, yellow, purple and pink for aerobic, butyrate, infant stool metabolites and anaerobic, respectively. The change in expression of each gene was evaluated using two criteria: only *padj* (*padj* < 0.1) and *padj* (*padj* < 0.1) together with log2foldchange (|LFC| > 1). Columns K and L indicate the number of conditions in which each gene's expression changed according to these filtering parameters. The column named “Prophage/integrative element?” contains information about the location on each gene: core indicates that the gene is encoded on the main chromosome NC_011601, and non-core indicates that the gene is encoded on one of the three plasmids (NC_011602, NC_011603 [pEAF], and EU580135.1). Other categories are encoded on NC_011601 and are listed according to Iguchi et al.^57^ Column AN “Variable in technical Vir^EPEC^ and Avir^EPEC^ aerobic cond.” contains “1” for genes variable in technical replicates under aerobic condition. Putative virulence genes in EPEC in the aerobic condition sheet: Putative virulence genes based on the comparison between Vir^EPEC^ and Avir^EPEC^ microcolonies in the aerobic growth condition (*padj* < 0.1 & log2FoldChange > 1).^54^ The genes highlighted in yellow were identified according to the stringent criterion (*padj* < 0.1 & log2FoldChange > 2). Core and non-core indicate if the gene is encoded on the main chromosome NC_011601 or on the NC_011603 plasmid, respectively. Putative virulence-related genes in EPEC identified in two or more of the tested growth conditions sheet: Virulence associated or putative virulence genes identified in two, three, or four of the tested growth conditions by Microcolony-seq in the comparison between Vir^EPEC^ and Avir^EPEC^ microcolonies. The growth condition were: aerobic LB, aerobic LB supplemented with butyrate, aerobic LB supplemented with infant stool metabolites, and anaerobic LB (*padj* < 0.1 & log2FoldChange > 1).^54^ In this sheet, we wanted to highlight the putative virulence genes identified by the direct comparison between the two subpopulations. The column indicating the conditions in which each gene was identified as putative virulent is highlighted in orange.

Table S3. Differential expression analysis between subpopulations of anaerobic Vir^EPEC^ microcolonies and their WGS data, related to Figures 3 and 4 Normalization of mapped reads and differential expression analyses were carried out by DESeq2.^54^ Fim ON vs. Fim OFF sheet: Included are genes that were statistically significantly changed (*padj* < 0.1)^54^ between the *fim* ON and *fim* OFF Vir^EPEC^ subpopulations according to PC1 in Figure 3B. Normalized read columns that belong to the *fim* ON and *fim* OFF microcolonies were marked in pink and grey, respectively. Hyper-flagellated Vir^EPEC^ vs. Non-flagellated Vir^EPEC^ sheet: Included are genes that were statistically significantly changed (*padj* < 0.1 & |log2FoldChange| > 1)^54^ between the Hyper-flagellated and Non-flagellated Vir^EPEC^ subpopulations according to PC2 in Figure 4A. Normalized reads columns that belong to the Hyper-flagellated and to the Non-flagellated microcolonies were marked in green and yellow, respectively. Additional variable genes sheet: Included are genes that were identified as variable genes in the anaerobic Vir^EPEC^ microcolonies. Genes that appeared as variable genes in the technical replicates of the aerobic Vir^EPEC^ and of the Avir^EPEC^ microcolonies were excluded. Genes that belong to the motility, chemotaxis, or to the *fim* operon were excluded since they were included in other sheets. Genes that are encoded on LEE4 operon were indicated. Hyper-flagellated Vir^EPEC^ vs. Avir^EPEC^ sheet: Differential comparison between Hyper-flagellated Vir^EPEC^ vs. Avir^EPEC^ anaerobic microcolonies. Included are genes that belong to the flagellar assembly. Normalized counts of microcolonies belonging to the Hyper-flagellated Vir^EPEC^ and the Avir^EPEC^ subpopulations under anaerobic conditions are presented. Basemean, log2FoldChange, and *padj* were calculated by DESeq2^54^ between these two subpopulations. The differential expression analysis showed that the Hyper-flagellated Vir^EPEC^ expresses the flagellar genes at an even higher level than the Avir^EPEC^ subpopulation. “All genes” sheet: Included are all genes in the EPEC genome and their normalized expression values (DESeq2) in all Vir^EPEC^ and the Avir^EPEC^ subpopulations under anaerobic conditions. WGS EPEC mutations sheet: *breseq*^122^ analysis of whole-genome sequencing for six Vir^EPEC^ and two Avir^EPEC^ anaerobic microcolonies determined only mutations in the *lrhA* gene in Vir^EPEC^ microcolonies 1, 3 and 5, leading to the Hyper-flagellated Vir^EPEC^ subpopulation. WGS EPEC phase variation sheet: Junction information from the breseq^122^ analysis of the WGS of six Vir^EPEC^ and two Avir^EPEC^ anaerobic microcolonies, representing phase-variation, was included. Vir^EPEC^ microcolonies 1, 5, and 11 were in the *fim* ON orientation, while Vir^EPEC^ microcolonies 3, 4, and 9 were in the *fim* OFF orientation. Two additional junctions, detected between genes *E2348C_2679* and *E2348C_2682*, showed similar frequencies in both Vir^EPEC^ and Avir^EPEC^ microcolonies and were previously reported by Goldberg et al.^73^

Table S4. Differential expression analysis between subpopulations of UPEC microcolonies obtained from a UTI, related to Figures 5 and 6 All genes appearing in the assembled genome were included in the table. Annotations were obtained using prokka.^124^ Normalization and differential expression were done using DESeq2.^54^ “Variable gene?” The value of ‘1’ appears for genes that appeared as dispersion outliers in the biological replicates only. “Basemean” is the normalized average expression among all 20 UTI microcolonies analyzed. Columns L, M and N include differential expression analysis between microcolonies separated along PC1 in Figure 5D (differential expression between Vir^UTI^ vs. Avir^UTI^). Columns O, P and Q include differential expression analysis between microcolonies separated along PC1 in Figure 5B (differential expression between microcolonies that include an antibiotics resistance cluster and microcolonies that lost the cluster). Columns R–AK include the normalized counts for each microcolony for each gene. Columns AL–AW include information from the literature.

Table S5. WGS analysis of UPEC microcolonies from the urine of a patient with a UTI, related to Figures 5 and 6 Whole-genome sequencing data files were analyzed using *breseq* with default parameters.^122^*gdtools* COMPARE was used to compare the output of all tested microcolonies. Seq id specifies the contig id in which the mutation was identified. Position, mutation, and gene description were indicated as well as separate columns for each analyzed microcolony. The GenBank file to which the sequencing files were mapped by *breseq* was generated from the Pacbio sequencing of one technical sample (UTI_30). To these assembled contigs, additional three contigs assembled from unmapped reads of UTI_17 containing the antibiotic resistance cluster and the siderophore operons were added (designated p1, p2 and p3).

Table S6. Differential expression analysis between subpopulations of *S. aureus* microcolonies obtained from a BSI and their WGS data, related to Figure 7 RNA-seq sheet: All genes appearing in the assembled genome of *S. aureus* from the bloodstream infection were included in the table. Normalization and differential expression were done using DESeq2.^54^ Columns I and J include the log2FoldChange and *padj* values^54^ of the differential expression analysis of Vir^BSI^ vs. Intermediate^BSI^ plus Avir^BSI^ subpopulations. Columns K and L include the log2FoldChange and *padj* values^54^ of the differential expression analysis of Intermediate^BSI^ vs. Avir^BSI^ subpopulations. Column M: The average expression (basemean) of each gene in all bloodstream infection (BSI) microcolonies. Columns N–AJ: The normalized counts by DESeq2^54^ for each BSI microcolony. Column AK: The value of ‘1’ appears for genes that appeared as dispersion outliers, i.e., variable genes. Columns AL–AR: Include the value of ‘1’ in case the specific gene is part of the indicated pathway. WGS Mutations sheet: Whole-genome sequencing (WGS) of 23 bloodstream infection (BSI) microcolonies was analyzed using *breseq*.^122^ Only five mutations were identified, none of which correlated with the separation into Vir^BSI^, Intermediate^BSI^, and Avir^BSI^ subpopulations. All genomes were mapped to the reference genome of microcolony BSI_22, which underwent de novo genome assembly.

Methods S1. Evaluation of the spontaneous switching rates between fim states, related to STAR Methods

## References

[1] Kumar A. (2021). The Complex Genetic Basis and Multilayered Regulatory Control of Yeast Pseudohyphal Growth. Annu. Rev. Genet..

[2] Lenhart B.A., Meeks B., Murphy H.A. (2019). Variation in Filamentous Growth and Response to Quorum-Sensing Compounds in Environmental Isolates of Saccharomyces cerevisiae. G3 (Bethesda).

[3] Recker M., Buckee C.O., Serazin A., Kyes S., Pinches R., Christodoulou Z., Springer A.L., Gupta S., Newbold C.I. (2011). Antigenic variation in Plasmodium falciparum malaria involves a highly structured switching pattern. PLoS Pathog..

[4] Novick A., Weiner M. (1957). Enzyme Induction as an All-or-None Phenomenon. Proc. Natl. Acad. Sci. USA.

[5] Casadesús J., Low D.A. (2013). Programmed heterogeneity: epigenetic mechanisms in bacteria. J. Biol. Chem..

[6] Ackermann M. (2015). A functional perspective on phenotypic heterogeneity in microorganisms. Nat. Rev. Microbiol..

[7] Davis K.M., Isberg R.R. (2016). Defining heterogeneity within bacterial populations via single cell approaches. BioEssays.

[8] Elowitz M.B., Levine A.J., Siggia E.D., Swain P.S. (2002). Stochastic gene expression in a single cell. Science.

[9] Golding I., Paulsson J., Zawilski S.M., Cox E.C. (2005). Real-time kinetics of gene activity in individual bacteria. Cell.

[10] Wang H., Palasik B.N. (2022). Combating antimicrobial resistance with cefiderocol for complicated infections involving the urinary tract. Ther. Adv. Urol..

[11] Roe A.J., Yull H., Naylor S.W., Woodward M.J., Smith D.G., Gally D.L. (2003). Heterogeneous surface expression of EspA translocon filaments by Escherichia coli O157:H7 is controlled at the posttranscriptional level. Infect. Immun..

[12] Balázsi G., van Oudenaarden A., Collins J.J. (2011). Cellular decision making and biological noise: from microbes to mammals. Cell.

[13] Beaumont H.J., Gallie J., Kost C., Ferguson G.C., Rainey P.B. (2009). Experimental evolution of bet hedging. Nature.

[14] Balaban N.Q., Merrin J., Chait R., Kowalik L., Leibler S. (2004). Bacterial persistence as a phenotypic switch. Science.

[15] Avraham R., Haseley N., Brown D., Penaranda C., Jijon H.B., Trombetta J.J., Satija R., Shalek A.K., Xavier R.J., Regev A. (2015). Pathogen Cell-to-Cell Variability Drives Heterogeneity in Host Immune Responses. Cell.

[16] Helaine S., Thompson J.A., Watson K.G., Liu M., Boyle C., Holden D.W. (2010). Dynamics of intracellular bacterial replication at the single cell level. Proc. Natl. Acad. Sci. USA.

[17] Vulin C., Leimer N., Huemer M., Ackermann M., Zinkernagel A.S. (2018). Prolonged bacterial lag time results in small colony variants that represent a sub-population of persisters. Nat. Commun..

[18] Blyn L.B., Braaten B.A., Low D.A. (1990). Regulation of pap pilin phase variation by a mechanism involving differential dam methylation states. EMBO J..

[19] Hernday A., Krabbe M., Braaten B., Low D. (2002). Self-perpetuating epigenetic pili switches in bacteria. Proc. Natl. Acad. Sci. USA.

[20] Moffitt J.R., Pandey S., Boettiger A.N., Wang S., Zhuang X. (2016). Spatial organization shapes the turnover of a bacterial transcriptome. eLife.

[21] Armbruster C.R., Lee C.K., Parker-Gilham J., de Anda J., Xia A., Zhao K., Murakami K., Tseng B.S., Hoffman L.R., Jin F. (2019). Heterogeneity in surface sensing suggests a division of labor in Pseudomonas aeruginosa populations. eLife.

[22] Rosenthal A.Z., Qi Y., Hormoz S., Park J., Li S.H., Elowitz M.B. (2018). Metabolic interactions between dynamic bacterial subpopulations. eLife.

[23] Arnoldini M., Vizcarra I.A., Peña-Miller R., Stocker N., Diard M., Vogel V., Beardmore R.E., Hardt W.D., Ackermann M. (2014). Bistable expression of virulence genes in salmonella leads to the formation of an antibiotic-tolerant subpopulation. PLoS Biol..

[24] Laventie B.J., Sangermani M., Estermann F., Manfredi P., Planes R., Hug I., Jaeger T., Meunier E., Broz P., Jenal U. (2019). A Surface-Induced Asymmetric Program Promotes Tissue Colonization by Pseudomonas aeruginosa. Cell Host Microbe.

[25] Reyes Ruiz L.M., Williams C.L., Tamayo R. (2020). Enhancing bacterial survival through phenotypic heterogeneity. PLoS Pathog..

[26] Schwan W.R. (2011). Regulation of fim genes in uropathogenic Escherichia coli. World J. Clin. Infect. Dis..

[27] Lederberg J., Iino T. (1956). Phase Variation in Salmonella. Genetics.

[28] Ozbudak E.M., Thattai M., Lim H.N., Shraiman B.I., Van Oudenaarden A. (2004). Multistability in the lactose utilization network of Escherichia coli. Nature.

[29] Leh H., Khodr A., Bouger M.C., Sclavi B., Rimsky S., Bury-Moné S. (2017). Bacterial-Chromatin Structural Proteins Regulate the Bimodal Expression of the Locus of Enterocyte Effacement (LEE) Pathogenicity Island in Enteropathogenic Escherichia coli. mBio.

[30] Ronin I., Katsowich N., Rosenshine I., Balaban N.Q. (2017). A long-term epigenetic memory switch controls bacterial virulence bimodality. eLife.

[31] Frankel G., Philips A.D., Novakova M., Batchelor M., Hicks S., Dougan G. (1998). Generation of Escherichia coli intimin derivatives with differing biological activities using site-directed mutagenesis of the intimin C-terminus domain. Mol. Microbiol..

[32] Tipton K.A., Dimitrova D., Rather P.N. (2015). Phase-Variable Control of Multiple Phenotypes in Acinetobacter baumannii Strain AB5075. J. Bacteriol..

[33] Chin C.Y., Tipton K.A., Farokhyfar M., Burd E.M., Weiss D.S., Rather P.N. (2018). A high-frequency phenotypic switch links bacterial virulence and environmental survival in Acinetobacter baumannii. Nat. Microbiol..

[34] Blevins S.M., Bronze M.S. (2010). Robert Koch and the 'golden age' of bacteriology. Int. J. Infect Dis..

[35] Blattman S.B., Jiang W., Oikonomou P., Tavazoie S. (2020). Prokaryotic single-cell RNA sequencing by in situ combinatorial indexing. Nat. Microbiol..

[36] Homberger C., Hayward R.J., Barquist L., Vogel J. (2023). Improved Bacterial Single-Cell RNA-Seq through Automated MATQ-Seq and Cas9-Based Removal of rRNA Reads. mBio.

[37] Imdahl F., Vafadarnejad E., Homberger C., Saliba A.E., Vogel J. (2020). Single-cell RNA-sequencing reports growth-condition-specific global transcriptomes of individual bacteria. Nat. Microbiol..

[38] Kuchina A., Brettner L.M., Paleologu L., Roco C.M., Rosenberg A.B., Carignano A., Kibler R., Hirano M., DePaolo R.W., Seelig G. (2021). Microbial single-cell RNA sequencing by split-pool barcoding. Science.

[39] Ma P., Amemiya H.M., He L.L., Gandhi S.J., Nicol R., Bhattacharyya R.P., Smillie C.S., Hung D.T. (2023). Bacterial droplet-based single-cell RNA-seq reveals antibiotic-associated heterogeneous cellular states. Cell.

[40] McNulty R., Sritharan D., Pahng S.H., Meisch J.P., Liu S., Brennan M.A., Saxer G., Hormoz S., Rosenthal A.Z. (2023). Probe-based bacterial single-cell RNA sequencing predicts toxin regulation. Nat. Microbiol..

[41] Wang B., Lin A.E., Yuan J., Novak K.E., Koch M.D., Wingreen N.S., Adamson B., Gitai Z. (2023). Single-cell massively-parallel multiplexed microbial sequencing (M3-seq) identifies rare bacterial populations and profiles phage infection. Nat. Microbiol..

[42] Nishimura M., Takeyama H., Hosokawa M. (2023). Enhancing the sensitivity of bacterial single-cell RNA sequencing using RamDA-seq and Cas9-based rRNA depletion. J. Biosci. Bioeng..

[43] Xu Z., Wang Y., Sheng K., Rosenthal R., Liu N., Hua X., Zhang T., Chen J., Song M., Lv Y. (2023). Droplet-based high-throughput single microbe RNA sequencing by smRandom-seq. Nat. Commun..

[44] Capp J.P. (2021). Interplay between genetic, epigenetic, and gene expression variability: Considering complexity in evolvability. Evol. Appl..

[45] Chen H.D., Frankel G. (2005). Enteropathogenic Escherichia coli: unravelling pathogenesis. FEMS Microbiol. Rev..

[46] Clarke S.C., Haigh R.D., Freestone P.P., Williams P.H. (2003). Virulence of enteropathogenic Escherichia coli, a global pathogen. Clin. Microbiol. Rev..

[47] Vallance B.A., Finlay B.B. (2000). Exploitation of host cells by enteropathogenic Escherichia coli. Proc. Natl. Acad. Sci. USA.

[48] Bingle L.E., Constantinidou C., Shaw R.K., Islam M.S., Patel M., Snyder L.A., Lee D.J., Penn C.W., Busby S.J., Pallen M.J. (2014). Microarray analysis of the Ler regulon in enteropathogenic and enterohaemorrhagic Escherichia coli strains. PLoS One.

[49] Hazen T.H., Daugherty S.C., Shetty A., Mahurkar A.A., White O., Kaper J.B., Rasko D.A. (2015). RNA-Seq analysis of isolate- and growth phase-specific differences in the global transcriptomes of enteropathogenic Escherichia coli prototype isolates. Front. Microbiol..

[50] Hazen T.H., Daugherty S.C., Shetty A.C., Nataro J.P., Rasko D.A. (2017). Transcriptional Variation of Diverse Enteropathogenic Escherichia coli Isolates under Virulence-Inducing Conditions. mSystems.

[51] Pearl Mizrahi S., Elbaz N., Argaman L., Altuvia Y., Katsowich N., Socol Y., Bar A., Rosenshine I., Margalit H. (2021). The impact of Hfq-mediated sRNA-mRNA interactome on the virulence of enteropathogenic Escherichia coli. Sci. Adv..

[52] Leverton L.Q., Kaper J.B. (2005). Temporal expression of enteropathogenic Escherichia coli virulence genes in an in vitro model of infection. Infect. Immun..

[53] Levin-Reisman I., Fridman O., Balaban N.Q. (2014). ScanLag: high-throughput quantification of colony growth and lag time. J. Vis. Exp..

[54] Love M.I., Huber W., Anders S. (2014). Moderated estimation of fold change and dispersion for RNA-seq data with DESeq2. Genome Biol..

[55] Shaffer S.M., Emert B.L., Reyes Hueros R.A., Cote C., Harmange G., Schaff D.L., Sizemore A.E., Gupte R., Torre E., Singh A. (2020). Memory Sequencing Reveals Heritable Single-Cell Gene Expression Programs Associated with Distinct Cellular Behaviors. Cell.

[56] Gaytán M.O., Martínez-Santos V.I., Soto E., González-Pedrajo B. (2016). Type Three Secretion System in Attaching and Effacing Pathogens. Front. Cell. Infect. Microbiol..

[57] Iguchi A., Thomson N.R., Ogura Y., Saunders D., Ooka T., Henderson I.R., Harris D., Asadulghani M., Kurokawa K., Dean P. (2009). Complete genome sequence and comparative genome analysis of enteropathogenic Escherichia coli O127:H6 strain E2348/69. J. Bacteriol..

[58] Little J.I., Singh P.K., Zhao J., Dunn S., Matz H., Donnenberg M.S. (2024). Type IV pili of Enterobacteriaceae species. EcoSal Plus.

[59] Martinez de la Peña C.F., De Masi L., Nisa S., Mulvey G., Tong J., Donnenberg M.S., Armstrong G.D. (2015). BfpI, BfpJ, and BfpK Minor Pilins Are Important for the Function and Biogenesis of Bundle-Forming Pili Expressed by Enteropathogenic Escherichia coli. J. Bacteriol..

[60] Du K., Bereswill S., Heimesaat M.M. (2021). A literature survey on antimicrobial and immune-modulatory effects of butyrate revealing non-antibiotic approaches to tackle bacterial infections. Eur. J. Microbiol. Immunol. (Bp).

[61] Hockenberry A.M., Micali G., Takács G., Weng J., Hardt W.D., Ackermann M. (2021). Microbiota-derived metabolites inhibit Salmonella virulent subpopulation development by acting on single-cell behaviors. Proc. Natl. Acad. Sci. USA.

[62] Machado M.G., Sencio V., Trottein F. (2021). Short-Chain Fatty Acids as a Potential Treatment for Infections: a Closer Look at the Lungs. Infect. Immun..

[63] Ochoa T.J., Contreras C.A. (2011). Enteropathogenic escherichia coli infection in children. Curr. Opin. Infect. Dis..

[64] Baldi D.L., Higginson E.E., Hocking D.M., Praszkier J., Cavaliere R., James C.E., Bennett-Wood V., Azzopardi K.I., Turnbull L., Lithgow T. (2012). The type II secretion system and its ubiquitous lipoprotein substrate, SslE, are required for biofilm formation and virulence of enteropathogenic Escherichia coli. Infect. Immun..

[65] Krekhno Z., Woodward S.E., Serapio-Palacios A., Peña-Díaz J., Moon K.M., Foster L.J., Finlay B.B. (2024). Citrobacter rodentium possesses a functional type II secretion system necessary for successful host infection. Gut Microbes.

[66] Goosney D.L., Gruenheid S., Finlay B.B. (2000). Gut feelings: enteropathogenic E. coli (EPEC) interactions with the host. Annu. Rev. Cell Dev. Biol..

[67] Peleg A., Shifrin Y., Ilan O., Nadler-Yona C., Nov S., Koby S., Baruch K., Altuvia S., Elgrably-Weiss M., Abe C.M. (2005). Identification of an Escherichia coli operon required for formation of the O-antigen capsule. J. Bacteriol..

[68] Ionescu M., Belkin S. (2009). Simple quantification of bacterial envelope-associated extracellular materials. J. Microbiol. Methods.

[69] Bustamante V.H., Villalba M.I., García-Angulo V.A., Vázquez A., Martínez L.C., Jiménez R., Puente J.L. (2011). PerC and GrlA independently regulate Ler expression in enteropathogenic Escherichia coli. Mol. Microbiol..

[70] Martínez-Laguna Y., Calva E., Puente J.L. (1999). Autoactivation and environmental regulation of bfpT expression, the gene coding for the transcriptional activator of bfpA in enteropathogenic Escherichia coli. Mol. Microbiol..

[71] Siegele D.A., Hu J.C. (1997). Gene expression from plasmids containing the araBAD promoter at subsaturating inducer concentrations represents mixed populations. Proc. Natl. Acad. Sci. USA.

[72] Connell I., Agace W., Klemm P., Schembri M., Mărild S., Svanborg C. (1996). Type 1 fimbrial expression enhances Escherichia coli virulence for the urinary tract. Proc. Natl. Acad. Sci. USA.

[73] Goldberg A., Fridman O., Ronin I., Balaban N.Q. (2014). Systematic identification and quantification of phase variation in commensal and pathogenic Escherichia coli. Genome Med..

[74] Adiciptaningrum A.M., Blomfield I.C., Tans S.J. (2009). Direct observation of type 1 fimbrial switching. EMBO Rep..

[75] Gally D.L., Bogan J.A., Eisenstein B.I., Blomfield I.C. (1993). Environmental regulation of the fim switch controlling type 1 fimbrial phase variation in Escherichia coli K-12: effects of temperature and media.. J. Bacteriol..

[76] Müller C.M., Aberg A., Straseviçiene J., Emody L., Uhlin B.E., Balsalobre C. (2009). Type 1 fimbriae, a colonization factor of uropathogenic Escherichia coli, are controlled by the metabolic sensor CRP-cAMP. PLoS Pathog..

[77] Zhou K., Aertsen A., Michiels C.W. (2014). The role of variable DNA tandem repeats in bacterial adaptation. FEMS Microbiol. Rev..

[78] Lehnen D., Blumer C., Polen T., Wackwitz B., Wendisch V.F., Unden G. (2002). LrhA as a new transcriptional key regulator of flagella, motility and chemotaxis genes in Escherichia coli. Mol. Microbiol..

[79] Parker D.J., Demetci P., Li G.W. (2019). Rapid Accumulation of Motility-Activating Mutations in Resting Liquid Culture of Escherichia coli. J. Bacteriol..

[80] Barreto H.C., Sousa A., Gordo I. (2020). The Landscape of Adaptive Evolution of a Gut Commensal Bacteria in Aging Mice. Curr. Biol..

[81] Kisiela D.I., Radey M., Paul S., Porter S., Polukhina K., Tchesnokova V., Shevchenko S., Chan D., Aziz M., Johnson T.J. (2017). Inactivation of Transcriptional Regulators during Within-Household Evolution of Escherichia coli. J. Bacteriol..

[82] Schneider C.A., Rasband W.S., Eliceiri K.W. (2012). NIH Image to ImageJ: 25 years of image analysis. Nat. Methods.

[83] Levin-Reisman I., Gefen O., Fridman O., Ronin I., Shwa D., Sheftel H., Balaban N.Q. (2010). Automated imaging with ScanLag reveals previously undetectable bacterial growth phenotypes. Nat. Methods.

[84] Robinson A.E., Heffernan J.R., Henderson J.P. (2018). The iron hand of uropathogenic Escherichia coli: the role of transition metal control in virulence. Future Microbiol..

[85] Bielecki P., Muthukumarasamy U., Eckweiler D., Bielecka A., Pohl S., Schanz A., Niemeyer U., Oumeraci T., von Neuhoff N., Ghigo J.M. (2014). In vivo mRNA profiling of uropathogenic Escherichia coli from diverse phylogroups reveals common and group-specific gene expression profiles. mBio.

[86] Nielsen K.L., Stegger M., Kiil K., Lilje B., Ejrnæs K., Leihof R.F., Skjøt-Rasmussen L., Godfrey P., Monsen T., Ferry S. (2021). Escherichia coli Causing Recurrent Urinary Tract Infections: Comparison to Non-Recurrent Isolates and Genomic Adaptation in Recurrent Infections. Microorganisms.

[87] Cassat J.E., Skaar E.P. (2013). Iron in infection and immunity. Cell Host Microbe.

[88] Chand U., Priyambada P., Kushawaha P.K. (2023). Staphylococcus aureus vaccine strategy: Promise and challenges. Microbiol. Res..

[89] Clegg J., Soldaini E., McLoughlin R.M., Rittenhouse S., Bagnoli F., Phogat S. (2021). Staphylococcus aureus Vaccine Research and Development: The Past, Present and Future, Including Novel Therapeutic Strategies. Front. Immunol..

[90] Bagnoli F., Bertholet S., Grandi G. (2012). Inferring reasons for the failure of Staphylococcus aureus vaccines in clinical trials. Front. Cell. Infect. Microbiol..

[91] Lamy B., Dargère S., Arendrup M.C., Parienti J.J., Tattevin P. (2016). How to Optimize the Use of Blood Cultures for the Diagnosis of Bloodstream Infections? A State-of-the Art. Front. Microbiol..

[92] Beetham C.M., Schuster C.F., Kviatkovski I., Santiago M., Walker S., Gründling A. (2024). Histidine transport is essential for the growth of Staphylococcus aureus at low pH. PLoS Pathog..

[93] Zhou C., Fey P.D. (2020). The acid response network of Staphylococcus aureus. Curr. Opin. Microbiol..

[94] Wilton M., Charron-Mazenod L., Moore R., Lewenza S. (2016). Extracellular DNA Acidifies Biofilms and Induces Aminoglycoside Resistance in Pseudomonas aeruginosa. Antimicrob. Agents Chemother..

[95] Fisher R.A., Gollan B., Helaine S. (2017). Persistent bacterial infections and persister cells. Nat. Rev. Microbiol..

[96] Putrinš M., Kogermann K., Lukk E., Lippus M., Varik V., Tenson T. (2015). Phenotypic heterogeneity enables uropathogenic Escherichia coli to evade killing by antibiotics and serum complement. Infect. Immun..

[97] Claudi B., Spröte P., Chirkova A., Personnic N., Zankl J., Schürmann N., Schmidt A., Bumann D. (2014). Phenotypic variation of Salmonella in host tissues delays eradication by antimicrobial chemotherapy. Cell.

[98] Ersoy S.C., Heithoff D.M., Barnes L.t., Tripp G.K., House J.K., Marth J.D., Smith J.W., Mahan M.J. (2017). Correcting a Fundamental Flaw in the Paradigm for Antimicrobial Susceptibility Testing. EBiomedicine.

[99] Kumaraswamy M., Lin L., Olson J., Sun C.F., Nonejuie P., Corriden R., Döhrmann S., Ali S.R., Amaro D., Rohde M. (2016). Standard susceptibility testing overlooks potent azithromycin activity and cationic peptide synergy against MDR Stenotrophomonas maltophilia. J. Antimicrob. Chemother..

[100] Sollier J., Basler M., Broz P., Dittrich P.S., Drescher K., Egli A., Harms A., Hierlemann A., Hiller S., King C.G. (2024). Revitalizing antibiotic discovery and development through in vitro modelling of in-patient conditions. Nat. Microbiol..

[101] Lim H.G., Gao Y., Rychel K., Lamoureux C., Lou X.A., Palsson B.O. (2025). Revealing systematic changes in the transcriptome during the transition from exponential growth to stationary phase. mSystems.

[102] Nyström T. (2004). Stationary-phase physiology. Annu. Rev. Microbiol..

[103] Haiko J., Westerlund-Wikström B. (2013). The role of the bacterial flagellum in adhesion and virulence. Biology (Basel).

[104] Dar D., Dar N., Cai L., Newman D.K. (2021). Spatial transcriptomics of planktonic and sessile bacterial populations at single-cell resolution. Science.

[105] Cummings L.A., Wilkerson W.D., Bergsbaken T., Cookson B.T. (2006). In vivo, fliC expression by Salmonella enterica serovar Typhimurium is heterogeneous, regulated by ClpX, and anatomically restricted. Mol. Microbiol..

[106] Lyu Z., Yang A., Villanueva P., Singh A., Ling J. (2021). Heterogeneous Flagellar Expression in Single Salmonella Cells Promotes Diversity in Antibiotic Tolerance. mBio.

[107] Morawska L.P., Hernandez-Valdes J.A., Kuipers O.P. (2022). Diversity of bet-hedging strategies in microbial communities-Recent cases and insights. Wires Mech. Dis..

[108] Giri S., Waschina S., Kaleta C., Kost C. (2019). Defining Division of Labor in Microbial Communities. J. Mol. Biol..

[109] Sobota M., Rodilla Ramirez P.N., Cambré A., Rocker A., Mortier J., Gervais T., Haas T., Cornillet D., Chauvin D., Hug I. (2022). The expression of virulence genes increases membrane permeability and sensitivity to envelope stress in Salmonella Typhimurium. PLoS Biol..

[110] Johnson E.E., Wessling-Resnick M. (2012). Iron metabolism and the innate immune response to infection. Microbes Infect..

[111] Flores-Mireles A.L., Walker J.N., Caparon M., Hultgren S.J. (2015). Urinary tract infections: epidemiology, mechanisms of infection and treatment options. Nat. Rev. Microbiol..

[112] Alteri C.J., Hagan E.C., Sivick K.E., Smith S.N., Mobley H.L. (2009). Mucosal immunization with iron receptor antigens protects against urinary tract infection. PLoS Pathog..

[113] McCarthy M.W. (2020). Cefiderocol to treat complicated urinary tract infection. Drugs Today (Barc).

[114] Blango M.G., Mulvey M.A. (2010). Persistence of uropathogenic Escherichia coli in the face of multiple antibiotics. Antimicrob. Agents Chemother..

[115] Jahantigh H.R., Faezi S., Habibi M., Mahdavi M., Stufano A., Lovreglio P., Ahmadi K. (2022). The Candidate Antigens to Achieving an Effective Vaccine against Staphylococcus aureus. Vaccines (Basel).

[116] Patti J.M. (2004). A humanized monoclonal antibody targeting Staphylococcus aureus. Vaccine.

[117] Ke S., Kil H., Roggy C., Shields T., Quinn Z., Quinn A.P., Small J.M., Towne F.D., Brooks A.E., Brooks B.D. (2024). Potential Therapeutic Targets for Combination Antibody Therapy Against Staphylococcus aureus Infections. Antibiotics (Basel).

[118] Varshney A.K., Kuzmicheva G.A., Lin J., Sunley K.M., Bowling R.A., Kwan T.Y., Mays H.R., Rambhadran A., Zhang Y., Martin R.L. (2018). A natural human monoclonal antibody targeting Staphylococcus Protein A protects against Staphylococcus aureus bacteremia. PLoS One.

[119] Fridman O., Goldberg A., Ronin I., Shoresh N., Balaban N.Q. (2014). Optimization of lag time underlies antibiotic tolerance in evolved bacterial populations. Nature.

[120] Kim J.M., Garcia-Alcala M., Balleza E., Cluzel P. (2020). Stochastic transcriptional pulses orchestrate flagellar biosynthesis in Escherichia coli. Sci. Adv..

[121] Homberger C., Barquist L., Vogel J. (2022). Ushering in a new era of single-cell transcriptomics in bacteria. Microlife.

[122] Deatherage D.E., Barrick J.E. (2014). Identification of mutations in laboratory-evolved microbes from next-generation sequencing data using breseq. Methods Mol. Biol..

[123] Wick R.R., Judd L.M., Gorrie C.L., Holt K.E. (2017). Unicycler: Resolving bacterial genome assemblies from short and long sequencing reads. PLoS Comput. Biol..

[124] Seemann T. (2014). Prokka: rapid prokaryotic genome annotation. Bioinformatics.

[125] Faigenbaum-Romm R., Reich A., Gatt Y.E., Barsheshet M., Argaman L., Margalit H. (2020). Hierarchy in Hfq Chaperon Occupancy of Small RNA Targets Plays a Major Role in Their Regulation. Cell Rep..

[126] Culviner P.H., Guegler C.K., Laub M.T. (2020). A Simple, Cost-Effective, and Robust Method for rRNA Depletion in RNA-Sequencing Studies. mBio.

[127] Shishkin A.A., Giannoukos G., Kucukural A., Ciulla D., Busby M., Surka C., Chen J., Bhattacharyya R.P., Rudy R.F., Patel M.M. (2015). Simultaneous generation of many RNA-seq libraries in a single reaction. Nat. Methods.

[128] Melamed S., Faigenbaum-Romm R., Peer A., Reiss N., Shechter O., Bar A., Altuvia Y., Argaman L., Margalit H. (2018). Mapping the small RNA interactome in bacteria using RIL-seq. Nat. Protoc..

[129] Andrews, S. (2010). FastQC: A Quality Control Tool for High Throughput Sequence Data. https://www.bioinformatics.babraham.ac.uk/projects/fastqc/.

[130] Martin M. (2011). Cutadapt removes adapter sequences from high-throughput sequencing reads. EMBnet J..

[131] Danecek P., Bonfield J.K., Liddle J., Marshall J., Ohan V., Pollard M.O., Whitwham A., Keane T., McCarthy S.A., Davies R.M. (2021). Twelve years of SAMtools and BCFtools. GigaScience.

[132] Li H., Durbin R. (2009). Fast and accurate short read alignment with Burrows-Wheeler transform. Bioinformatics.

[133] Felsenstein J. (1989). PHYLIP -- Phylogeny Inference Package, [Version 3.2]. Cladistics.

[134] Boc A., Diallo A.B., Makarenkov V. (2012). T-REX: a web server for inferring, validating and visualizing phylogenetic trees and networks. Nucleic Acids Res..

[135] Edelstein A.D., Tsuchida M.A., Amodaj N., Pinkard H., Vale R.D., Stuurman N. (2014). Advanced methods of microscope control using muManager software. J. Biol. Methods.

[136] Pearl Mizrahi S., Gefen O., Simon I., Balaban N.Q. (2016). Persistence to anti-cancer treatments in the stationary to proliferating transition. Cell Cycle.

[137] Jumper J., Evans R., Pritzel A., Green T., Figurnov M., Ronneberger O., Tunyasuvunakool K., Bates R., Žídek A., Potapenko A. (2021). Highly accurate protein structure prediction with AlphaFold. Nature.

