# Supplementary material for: Uncovering phenotypic inheritance from single cells with Microcolony-seq
Source: Cell. 2025 Sep 18;188(19):5313–5331.e18. doi: 10.1016/j.cell.2025.08.001 (PMC12456302; doi:10.1016/j.cell.2025.08.001)
Supplement: Methods S1. Evaluation of the spontaneous switching rates between fim states, related to STAR Methods [file mmc7.pdf]

Methods S1 (related to STAR Methods)

Evaluation of the spontaneous switching rates between *fim* states

The spontaneous switching between the two states, "*fim* ON" and "*fim* OFF" during growth can be described by:

Eq. (2)
$$\begin{cases} \frac{dN^{ON}}{dt} = \mu N^{ON} - \gamma N^{ON} + \beta N^{OFF} \\ \frac{dN^{OFF}}{dt} = \mu N^{OFF} + \gamma N^{ON} - \beta N^{OFF} \end{cases}$$

Where N^{ON} and N^{OFF} are the number of bacteria in the ON and OFF states, respectively and μ is the growth rate of the population. γ is the switching rate from ON to OFF, and β the switching rate from OFF to ON.

According to the whole genome sequencing coverage and PCR, microcolonies in the OFF state contain no detectable ON bacteria, i.e. less than 1% of bacteria in the ON state. Therefore, we simplified Eq. (2) using $\beta=0$. In order to estimate an upper bound for γ, we used the whole genome coverage results, showing a fraction between 6-13% of OFF bacteria within the ON microcolony after 15 hours of growth with doubling time of 30 min. The solution of Eq. (2) with $\beta=0$ and leads to

Eq. (3)
$$\frac{N^{OFF}}{N^{OFF}+N^{ON}} = (1 - e^{-\gamma t})$$

The measured fraction of OFF bacteria within ON microcolonies results in γ between $2\text{x}10^{-3}$ to $5\text{x}10^{-3}$ per generation, i.e. orders of magnitude lower than previous estimates for *E. coli* K-12.[1]

Evaluation of the upper bound for the stability of inheritance detectable by Microcolony-seq:

The detection of distinct inherited phenotypes (named here as ON and OFF but applies to all bimodalities) in microcolonies depends on:

1) The rate of switching between phenotypes: γ is the switching rate from ON to OFF, and β the switching rate from OFF to ON.
2) The differential expression of the genes in the two phenotypes: g_{ON}, g_{OFF}
3) The growth rates of the two phenotypes

Assuming the worst scenario of only one gene differentially expressed between the two phenotypes, and with a differential expression ratio of log2 fold= 1.3, i.e., $g_{ON}/g_{OFF}=2.5$, we compute below the required stability of inheritance, i.e. the lowest rate of switching that would allow detection by Microcolony-seq, if a log2 fold difference of 1 is required:

The differential expression of the gene in the two microcolonies is required to be above 2 i.e.

$$\frac{G_{ON}}{G_{OFF}} > 2$$

$$\frac{G_{ON}}{G_{OFF}} = \frac{N(1-x)g_{ON} + Nxg_{OFF}}{N(1-x)g_{OFF} + Nxg_{ON}}$$

Where N is the total number of bacteria in a microcolony and x is the fraction of bacteria that have switched to the alternate phenotype within each colony. Our goal is first to evaluate the upper bound on x: using Eq. 1 and 2 and lower bound g_{ON}/g_{OFF}=2.5 we find

$$\frac{G_{ON}}{G_{OFF}} = \frac{2.5 - 1.5x}{1 + 1.5x} > 2$$

Leading to

$$0.1 > x$$

This requires the subpopulations of switched phenotypes to remain below 10% of the total population of the microcolony. In order to translate this percentage to an upper bound for the rate of switching, we solve the population dynamics using Eq. (3) for a colony initiated by a single bacterium while switching between two alternate phenotypes at rate γ leads to an upper bound of:

$$\gamma t < 0.14$$

as a microcolony consists of approximately 10^6 bacteria which are reached in 20 generations, we get $\gamma < 7.10^{-3}$ per generation. Note that we have assumed here that only one gene is differentially expressed, and at the limit of the required significance (log2 fold change of 1.3). In reality, many genes are differentially expressed between phenotypes, and their differential expression reaches log2 fold changes which are significantly higher than 1.3. Assuming a log2fold change of 2 increases the upper bound for γ to $2.\ 10^{-2}$ per generation. Therefore, we estimate that phenotypes with rates of switching in this range to be detectable by Microcolony-seq. In summary, the upper bound for the switching rate between states for the detection of inherited bimodality with Microcolony-seq is about 10^{-2} per generation and can be even higher if the differentially expressed genes ratio between the two phenotypes is significantly higher than 2. Therefore, the detectability of many bistable additional phenotypes should be possible.

References

1. Gally, D.L., Bogan, J.A., Eisenstein, B.I. and Blomfield, I.C. (1993). Environmental regulation of the fim switch controlling type 1 fimbrial phase variation in Escherichia coli K-12: effects of temperature and media. *J Bacteriol* 175, 6186-6193
